# Complementary biomolecular coassemblies direct energy transport for cardiac photostimulators

**DOI:** 10.1073/pnas.2509467122

**Published:** 2025-09-04

**Authors:** Ze-Fan Yao, Sujeung Lim, Yuyao Kuang, Emil M. Lundqvist, Natalie Celt, Caleb O. Chung, Kathryn K. Lee, Krystal Nguyen, Lanie Le, Sheng Wei Tang, Griffin M. Milligan, Phillip Kohl, Tarunya Rao Sudarshan, Youli Li, Asuka Eguchi, Anant K. Paravastu, Michael V. Zaragoza, Dmitry A. Fishman, Herdeline Ann M. Ardoña

**Affiliations:** ^a^Department of Chemical and Biomolecular Engineering, Samueli School of Engineering, University of California, Irvine, CA 92697; ^b^Department of Chemistry, School of Physical Sciences, University of California, Irvine, CA 92697; ^c^Department of Biomedical Engineering, Samueli School of Engineering, University of California, Irvine, CA 92697; ^d^Materials Research Laboratory and BioPolymers, Automated Cellular Infrastructure, Flow, and Integrated Chemistry Materials Innovation Platform (BioPACIFIC MIP), University of California, Santa Barbara, CA 93106; ^e^School of Chemical and Biomolecular Engineering, Georgia Institute of Technology, Atlanta, GA 30332; ^f^Department of Physiology and Biophysics, School of Medicine, University of California, Irvine, CA 92617; ^g^Sue and Bill Gross Stem Cell Research Center, University of California, Irvine, CA 92697; ^h^Parker H. Petit Institute for Bioengineering and Biosciences, Georgia Institute of Technology, Atlanta, GA 30332; ^i^Departments of Pediatrics and Biological Chemistry, School of Medicine, Irvine, CA 92617

**Keywords:** photostimulation, cardiac tissue engineering, self-assembly, peptide nanostructures, biomaterials

## Abstract

Charge and energy transport within living systems are fundamental processes that enable the autonomous function of excitable cells and tissues. To date, localized control of these transport processes has been enabled by genetic modification approaches to render light sensitivity to cells. Here, we present peptidic nanoassemblies as constituents of a cardiac biomaterial platform that leverages complementary sequence interactions to direct photoinduced energy transport at the cellular interface. Photophysical characterizations and conductivity measurements confirm the occurrence of energy/charge transfer and photocurrent generation upon optical excitation in both dry and electrolytic environments. Comparing an electrostatic sequence pair against a sequence-matched donor–acceptor coassembly, we demonstrate that the sequence design with charge complementarity shows more prominent photocurrent behavior. With the flanking bioadhesive units, the primary and stem cell–derived cardiomyocytes interfaced with covalently stabilized films of the optoelectronic nanostructures exhibited material-stimulated genotypic, structural, or functional cardiac features. Collectively, our findings introduce an optoelectronic cardiac biomaterial where coassembled peptide nanostructures are molecularly designed to induce light sensitivity in excitable cells without gene modification, influencing in vitro cardiac contractile behavior and expression of cardiac markers.

## Significance

Sustained electrophysiological signals play a critical role in powering the autonomous function of excitable tissues, such as those found in the heart. Traditional approaches to mimicking this phenomenon and delivering external electrical signals in vitro have been limited by the spatial resolution, specificity, and compatibility with soft interfaces due to the nature of the electrodes used in this process. Inspired by transport mechanisms in natural photosynthetic systems, here, we introduce a cardiac biomaterial interface composed of complementary peptide pairs that drive the ordering of electroactive units, serving as conduits for photoinduced energy transport. We show that light can be converted into cardiac stimulatory cues by synthetic biomacromolecules, with properties sensitive to the choice of sequence pairs.

Cells function as autonomous yet interconnected biological units that relay and receive signals from their environment. For excitable cells, such as cardiomyocytes that power the heart muscles, these signals include electric fields that mainly emerge from a coordinated flow of ions across the intra- and extracellular spaces (1, 2). Controlling the delivery of biophysical stimuli at the interface of excitable cells and tissues enables external modulation of their physiological functions (3–6). Current stimulation approaches, based on mechanical or electrical mechanisms, can regulate the development, maturation, and contraction/relaxation of cardiomyocytes and the tissues they form (2, 7–9). Applying external electrical fields through conventional electrode contacts, or via other synthetic interfaces more recently termed as electroceutical devices, have been used in vitro and in vivo to better study or control the contractile and electrophysiological functions of cardiomyocytes/cardiac tissues (10–15). While traditional electrode-based stimulation of cardiomyocytes is an effective way to regulate cellular and tissue behaviors, its spatiotemporal resolution limits its applicability for cell-specific mechanistic studies. These traditional stimulation methods are also based on rigid electrodes that do not recapitulate the natural mechanics of local cardiac microenvironments. Optogenetics currently stands as the state-of-the-art method for electrodeless, high-resolution stimulation of cardiac tissues in vitro; however, its main limitation lies in the species dependency of the gene modifications required to render cells light-sensitive (16–19). To this end, parallel efforts that leverage synthetic materials to precisely regulate cardiac behavior with fine spatiotemporal control or target specific cells remain highly sought after (20–26). These previous reports rely on synthetic inorganic, polymeric, or small molecule transducers. In this work, we present a biological photostimulatory system that is biomolecular in nature—hence, carrying the inherent potential of substrate patternability and sequence-specific properties or cellular interaction modes.

Biomolecular nanoassemblies in natural living systems, as well as those that exist in extracellular milieus, are known for their precision in building functional constructs and nanomaterials with properties that are highly dependent on the hierarchical molecular organization (27–31). Recent advances in the design of synthetic biomimetic systems have demonstrated sequence-dependent hierarchical biomolecule organization that can be exploited to control the assembly of functional materials (32–36). Peptides have emerged as versatile building blocks for nanostructure assembly due to their intrinsic biocompatibility, self-assembling ability, and programmability via sequence design of amino acids (37–40). The ability to control molecular ordering within synthetic peptide assemblies opens up new avenues for developing water-processable, biocompatible materials for bioelectronics and light-triggered biological modulation. Inspired by the protein-driven spatial organization of pigments in photosynthetic systems, the use of peptide segments for energy and charge transport at cell interfaces presents a viable approach for assembling energy-/charge-transporting π-conjugated systems. Here, complementary interactions between multiple peptide sequences (41–43) are utilized as part of the molecular engineering strategy to drive the formation of highly ordered structures of multiple π-electron systems with order-dependent transport efficiencies designed as a stimulatory cardiac biomaterial.

Donor–acceptor coassembly of organic π-conjugated molecules is widely used to enhance photocurrent generation, benefiting from controlled supramolecular order that improves power conversion efficiency in organic photovoltaic devices (44–48). This approach leverages the strategic combination of donor and acceptor materials to form a blended active layer, optimizing photophysical processes for the generation of effective charge carriers upon light illumination. This could serve as a photocurrent-generating material to remotely control electrophysiological signaling in excitable cardiac systems. In such a scheme, the donor materials, typically electron-rich π-conjugated molecules, can absorb photons and generate excitons, whereby excitons can migrate to the donor–acceptor interface and generate photocurrents after charge separation. The acceptor materials, typically electron-deficient π-conjugated molecules, can facilitate the transfer of electrons from the donor and benefit the formation of free charge carriers that contribute to the photocurrent (49–51). The intimate mixing of donor and acceptor molecules on the nanoscale is necessary to ensure that the generated carriers can efficiently reach the interface, thereby reducing recombination losses and enhancing the overall photocurrent generation performance. In the field of organic photovoltaics, molecular materials such as perylene diimide (PDI) and oligothiophenes are often utilized due to their favorable electronic properties and strong optical absorption in the visible range (52–54). The coassembly of such materials into the larger structures allows the separation of photoexcited carriers, ultimately enhancing energy conversion efficiency in organic photovoltaics (51, 55–57). These approaches are challenging to translate under aqueous, biologically relevant environments, such as those used in cell culture. Yet, the use of organic bioelectronic interfaces offers significant advantages, especially from a biomimicry perspective.

**Fig. 1.**
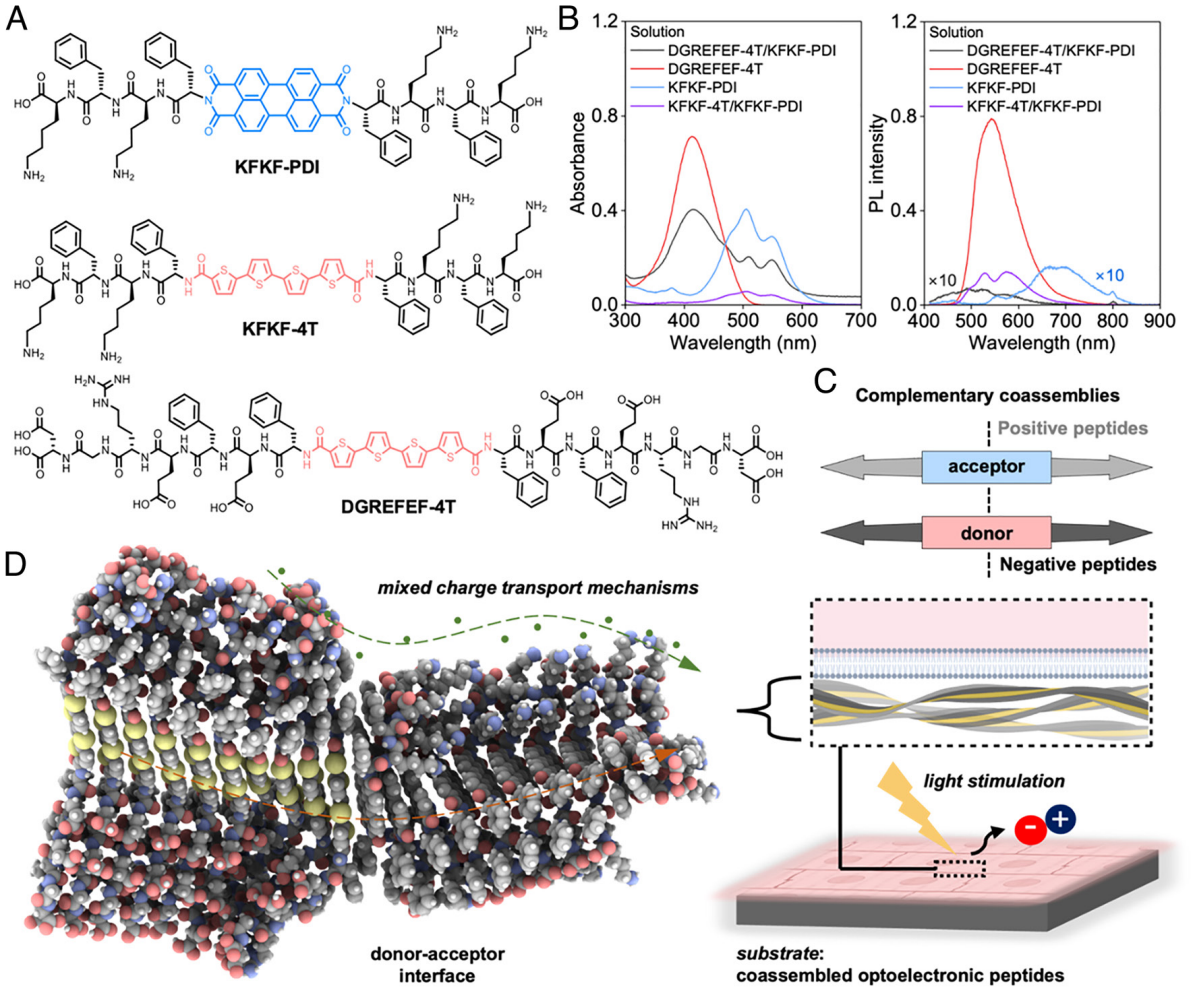
Photocurrent-generating coassembly of π-conjugated peptides as a cellular photostimulation platform. (*A*) Chemical structures of designed peptides of KFKF-PDI, KFKF-4T, and DGREFEF-4T. (*B*) Absorption and photoluminescence (PL) spectra of coassembly system (*λ*_exc_ = 400 nm). (*C*) Schematic for donor–acceptor coassembly with charge-complementary peptide sequence. (*D*) Optimized geometry of the coassembly between DGREFEF-4T (ten molecules on the left) and KFKF-PDI (ten molecules on the right) and mixed charge transport with light stimulation; schematic of the cellular photostimulation platform based on photocurrent-generating peptide assemblies.

In this work, we present a biomolecule-based stimulation platform for cardiac cells and tissues through optoelectronic peptides with sequence-controlled self-assembly and photophysical properties of the resulting nanostructures and bulk interfaces. Specifically, we direct donor–acceptor interactions within nanostructures by leveraging charge-complementary coassembly strategies to enable controlled nanoassembly and photostimulatory abilities upon interfacing with cardiac cells and tissues. The π-electron units, PDI and quaterthiophene (4T), are selected as the π-conjugated molecular core due to their n-type and p-type characteristics in charge transport, respectively (Fig. 1*A*). The aqueous nanostructure formation between these π-conjugated units is driven here via electrostatic coassembly between positive and negative charge-complementary peptide sequences, specifically DGREFEF and KFKF (from C to N termini). This sequence-driven approach for coassembling energy donor–acceptor units was designed to create a nanostructured environment that enables donor and acceptor molecules to interact effectively (Fig. 1 *B* and *C* and *SI Appendix*, Fig. S1) (58, 59), facilitating an efficient charge transfer and photophysical response upon light exposure (60). The incorporation of charged peptide sequences guarantees good solubility in aqueous solutions and promotes self-organization and stability of the nanostructures, ensuring that the donor and acceptor molecules are in proximity for optimal interaction and photocurrent generation. Furthermore, these designed π-conjugated peptides could be blended with established biopolymeric hydrogelators, such as gelatin, and can be patterned with microgrooved features to provide topological cues for inducing anisotropy of seeded cardiomyocytes as observed in healthy myocardial tissues in vivo. To demonstrate functional outcomes of interfacing coassembled π-conjugated peptide materials with cardiac systems *in vitro,* we show that the engineered biomolecular coassembly can influence the expression of cardiac markers of differentiated human induced pluripotent stem cell–derived cardiomyocytes (hiPSC-CMs), calcium handling behavior of neonatal rat ventricular myocytes (NRVMs), and in vitro cardiac monolayer contractions upon pulsed (modulated) light irradiation (Fig. 1*D*). In summary, we used complementary biomolecular interactions to order organic functional nanostructures, enabling the development of a paradigm for biomolecular material-based cardiac photostimulation.

## Results

To achieve cardiac photostimulation via optoelectronic peptide pairs, we designed two π-conjugated peptides (Fig. 1*A*) based on the principles of donor–acceptor and electrostatic complementarity (43). To create nanostructures that generate photocurrents under aqueous conditions, we designed π-conjugated peptides by integrating a p-type (4T) and another n-type (PDI) organic semiconductor with charge-complementary peptide sequences. The designed peptide sequences are DGREFEF and KFKF (from C to N termini) for producing DGREFEF-4T and KFKF-PDI, possessing net negative and positive charges under neutral pH conditions (58, 61). The energy donor unit bears an RGD peptide segment, a well-known integrin-binding fragment from fibronectin that is commonly used to enhance cell adhesion and direct cell patterning (62, 63). All functionalized peptides were successfully synthesized using standard solid-phase peptide synthesis and on-resin coupling with carboxylated π-conjugated units (*SI Appendix*, Figs. S2–S7). Due to ionizable residues spread across the aromatic units of each π-conjugated peptide, donor–acceptor, and charge-complementary interactions between 4T and PDI and negative/positive charges facilitate the coassembly under neutral cell culture conditions and photocurrent-generating properties (Fig. 1 *B* and *C*). To characterize the photophysical properties, the samples of both designed peptides were prepared by directly dissolving the purified sample in UltraPure water, where the coassembly occurred by mixing the peptide solutions with a 1:1 molar ratio. Absorption and photoluminescence spectra (Fig. 1*B*) suggest formation of coassembly between DGREFEF-4T and KFKF-PDI, where the photoluminescence intensity of coassembly is significantly diminished as compared to individual moieties (DGREFEF-4T and KFKF-PDI) in solutions. The observed fluorescence quenching supports the occurrence of energy transfer between donor and acceptor moieties. To serve as a reference point, a set of sequence-matched peptides of KFKF-4T and KFKF-PDI with the same KFKF peptide side chains and the same positive charges were designed to compare with the charge-complementary strategy. The mixtures of KFKF-4T and KFKF-PDI have shown smaller photoluminescence quenching compared with DGREFEF-4T and KFKF-PDI coassemblies, even though the absorption of the sequence-matched pairs was lower (Fig. 1*B*).

**Fig. 2.**
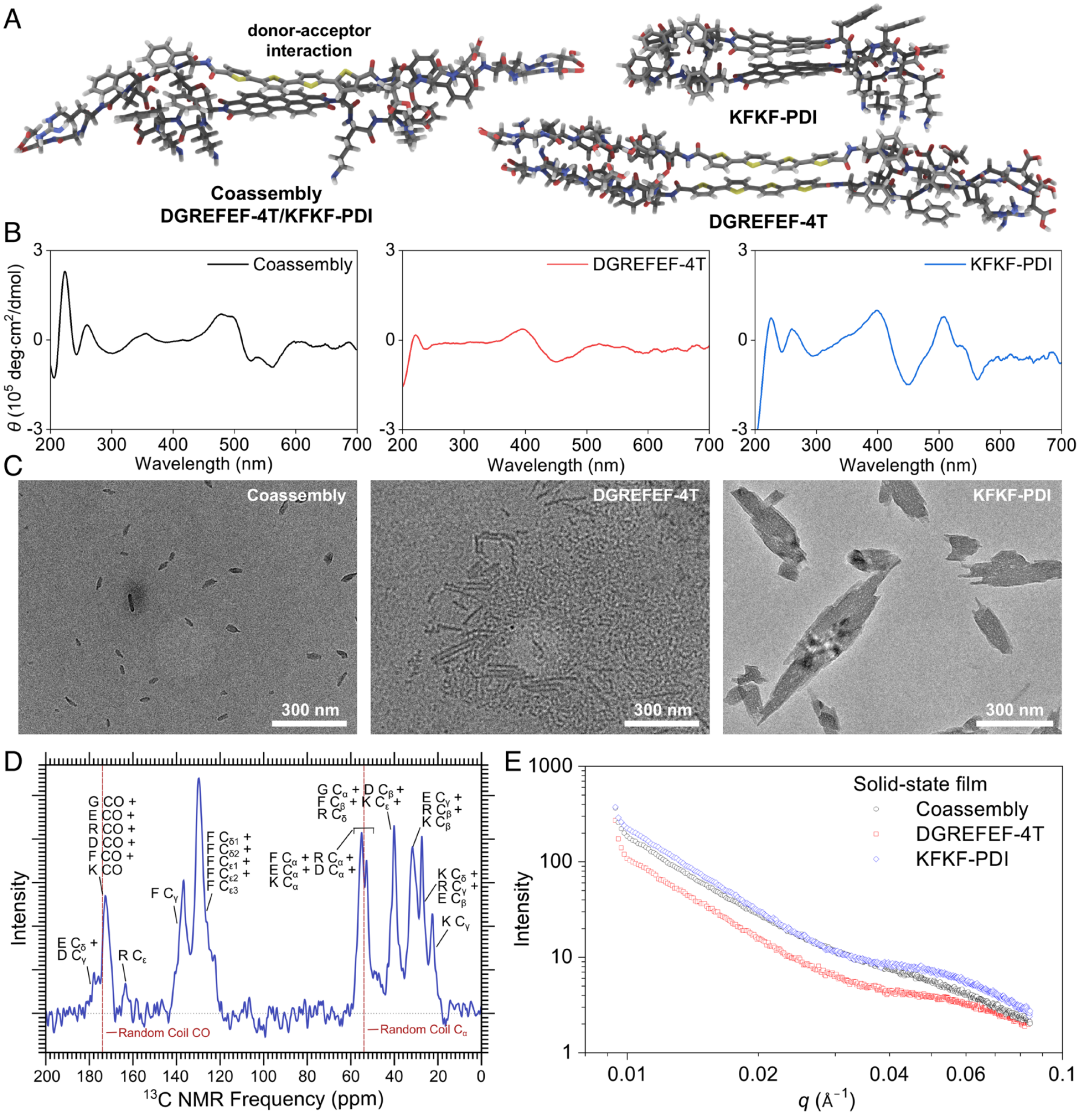
Self-assembly of π-conjugated peptides from molecules, solution state, to solid state. (*A*) Optimized dimer geometry of coassembly system. (*B*) Circular dichroism (CD) spectra of both individual and coassembly systems. (*C*) Transmission electron microscope (TEM) images of both individual and coassembly systems. (Scale bar, 300 nm.) (*D*) ^13^C solid-state NMR spectrum (natural abundance) of the coassembly. (*E*) Grazing-incidence small-angle X-ray scattering (GISAXS) of solid-state films of both individual and coassembly systems.

Next, we built the dimer stacking models of coassembly, DGREFEF-4T, and KFKF-PDI, respectively, and then performed structure optimizations using a semiempirical, tight-binding method (Fig. 2*A*) (64). The optimized dimer models showed π−π stacking and hydrogen bonding interactions, where the slightly slipped stacking corresponded well with the chiral signatures experimentally observed for these supramolecular coassemblies. Specifically, the circular dichroism (CD) spectra (Fig. 2*B* and *SI Appendix*, Fig. S8) around 400−600 nm indicate the stacking of π-conjugated units immersed in peptidic chiral environments (<250 nm). The nanostructures formed by these peptides were visualized using transmission electron microscopy (TEM) as shown in Fig. 2*C*. Compared with the nanostructures individually formed by DGREFEF-4T and KFKF-PDI, the coassembly of DGREFEF-4T and KFKF-PDI showed smaller dimensions and shorter aspect ratios. In addition, the nanostructures formed using DGREFEF-4T/KFKF-PDI and KFKF-4T/KFKF-PDI were visualized using confocal microscopy and high-magnification optical microscopy (*SI Appendix*, Figs. S9 and S10). The coassembly of DGREFEF-4T/KFKF-PDI displayed visible nanostructures while KFKF-4T/KFKF-PDI had minimal aggregates, distinguishing the influence of charge complementary interactions in facilitating the formation of coassembly and nanostructures. Solid-state NMR of natural-abundance ^13^C signal was performed to better understand the secondary structure and structural order within the coassembly, where the coassembled peptide showed *β*-sheet characteristics (Fig. 2*D*). To guide visual inspection, the signals of the amino acid weighted average of the expected carbonyl and *α*-carbon positions for the random coil structure were highlighted in red lines. The ^13^C solid-state NMR results indicate that all the peaks can be partially assigned to an amino acid near the expected *β*-sheet position for coassembly (65, 66). The ^13^C NMR line widths are consistent with an ordered peptide assembly (67–70). The carbonyl peak significantly shifted compared to the expected random-coil position in directions consistent with *β*-strand secondary structure. In contrast, we did not observe NMR signals for individual self-assemblies, indicating that the amounts of assembled material from the individual charged components were much less than for the coassembled samples. Furthermore, grazing-incidence small-angle X-ray scattering (GISAXS) of films formed from the coassembly system was performed to reveal the structural ordering of the peptide assemblies in the solid state (Fig. 2*E*). DGREFEF-4T and KFKF-PDI individual assemblies showed a broader scattering peak at the scattering vector (*q*) of around 0.055 Å^−1^, which corresponds to 114 Å of the structural sizes. With this, the coassembly of DGREFEF-4T and KFKF-PDI is expected to promote efficient photocurrent generation, where donor–acceptor interactions can facilitate the transport of photogenerated carriers. Consequently, attaching hydrophilic peptide sequences to the periphery of π-conjugated units enables their interaction with the surrounding aqueous environment, which not only helps interaction with living cells but also could aid in mixed charge transport of electrons and ions that can be implicated during cellular stimulations.

**Fig. 3.**
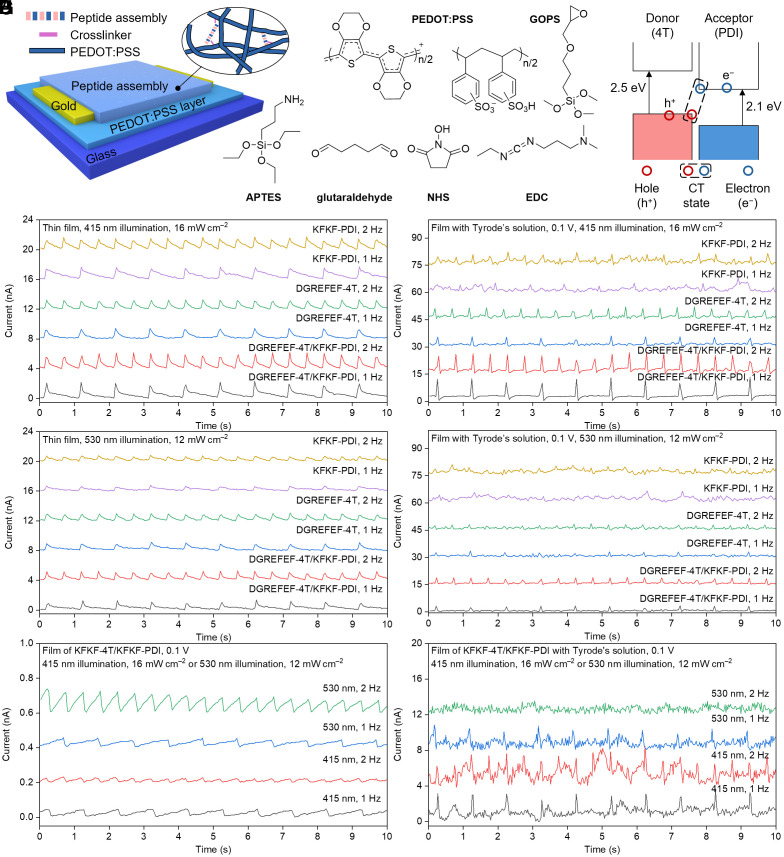
Photocurrent generation measurements for peptide-based interfaces. (*A*) Schematic diagram for the film structure of peptide assemblies on a conductive polymer layer. (*B*) Energy level diagram of donor and acceptor molecules showing charge generation process and the optical gaps calculated from the onset of absorption spectra. Photocurrent of peptide assemblies in (*C*) solid-state film and (*D*) Tyrode’s solution under illumination of 415 nm. Photocurrent of peptide assemblies in (*E*) solid-state film and (*F*) Tyrode’s solution under illumination of 530 nm. Photocurrent of KFKF-4T/KFKF-PDI in (*G*) solid-state film and (*H*) Tyrode’s solution under illumination of 415 and 530 nm.

The photocurrent generation properties of the peptide films were studied under various conditions, including dry film phases and aqueous environments (Tyrode’s solution) to mimic physiological conditions. First, we measured the photocurrents of the peptide films made by drop-casting directly onto a conductive layer poly(3,4-ethylenedioxythiophene) polystyrene sulfonate (PEDOT:PSS) crosslinked by (3-glycidyloxypropyl)trimethoxysilane (GOPS). To enhance the stability of the peptide films in aqueous environments, we employed a crosslinking method to bind more peptides to the substrate (Fig. 3 and *SI Appendix*, Fig. S11). Specifically, (3-aminopropyl)triethoxysilane (APTES) and glutaraldehyde were used to link KFKF-PDI onto the PEDOT:PSS layer, while APTES, 1-ethyl-3-(3-dimethylaminopropyl)carbodiimide (EDC) and N-hydroxysuccinimide (NHS) were utilized to covalently bond DGREFEF-4T to the PEDOT:PSS layer. GOPS was used to crosslink the PEDOT:PSS layer. This method was individually optimized for each peptide as well as for their coassembly (*SI Appendix*, Fig. S11). Following this surface linking procedure, both individual and coassembled peptides covalently adhered to the PEDOT:PSS layer, withstanding three cycles of washing with phosphate buffered saline (PBS) and UltraPure water. The long-term stability of the peptide films in aqueous conditions was confirmed using fluorescence imaging (*SI Appendix*, Fig. S12). The fluorescence signals remained stable and homogeneous across the samples after multiple washings and prolonged immersion in PBS (*SI Appendix*, Fig. S12). This suggests that peptides achieved stable covalent linkages on the PEDOT:PSS-coated glass substrates. After the deposition of peptides, the excess that was not crosslinked was washed off to ensure that the subsequent characterizations were from the adhered peptides. Both the coassembled and individual peptide molecules generated measurable photocurrent responses under both dry and wet conditions (Fig. 3 *C*–*H*). These measurements were done at 415 or 530 nm pulsed photoexcitation (donor and acceptor excitation, respectively) at 0.5 to 2 Hz pulsed light modulation frequencies, under both dry and wet conditions. The photocurrent values in the aqueous environment were notably higher than those measured in the dry film phase, suggesting the ionic contributions from Tyrode’s solution as an electrolytic medium.

Sequence-based donor−acceptor interfacing is beneficial to the charge separation and the photocurrent generation process, where the coassembly of DGREFEF-4T/KFKF-PDI here presents a viable donor−acceptor system as shown in the energy level diagram and inherent optical gaps of the selected π-electron cores (Fig. 3*B*) (71, 72). Consequently, coassembly returned photocurrent peak values of 2.14 ± 0.20 and 1.02 ± 0.02 nA (average ± SEM) under the illumination of 415 nm (16 mW cm^−2^, Fig. 3*C*) and 530 nm (12 mW cm^−2^, Fig. 3*E*). The individual components showed photocurrents of 1.20 ± 0.02 and 0.97 ± 0.02 nA in DGREFEF-4T under illumination of 415 and 530 nm, as well as 1.47 ± 0.04 and 0.92 ± 0.02 nA in KFKF-PDI under illumination of 415 and 530 nm, respectively. The blend of KFKF-4T/KFKF-PDI demonstrated a significantly lower photocurrent of 0.0233 ± 0.0008 and 0.0873 ± 0.003 nA under the illumination of 415 and 530 nm, respectively (Fig. 3*G*). DGREFEF-4T alone also exhibited a notable photocurrent, likely due to its efficient covalent bonding to the PEDOT:PSS substrate. However, when tested in Tyrode’s solution, the photocurrent behavior shifted (Fig. 3 *D* and *F*). In this aqueous environment, the coassembly of DGREFEF-4T/KFKF-PDI showed photocurrent peak values of 8.22 ± 0.30 and ~3 nA under the illumination of 415 and 530 nm, respectively. Meanwhile, the individual components showed photocurrent of 4.41 ± 0.16 and 2.17 ± 0.09 nA in DGREFEF-4T under the illumination of 415 and 530 nm, as well as 3.44 ± 0.32 and 2.71 ± 0.18 nA in KFKF-PDI under illumination of 415 and 530 nm, respectively. On the contrary, when compared with the blend of KFKF-4T/KFKF-PDI, the sequence-matched sample demonstrated a considerably lower photocurrent of 2.60 ± 0.01 and 0.71 ± 0.05 nA under the illumination of 415 and 530 nm, respectively, as compared to the pair with charge complementarity (Fig. 3*H*). Overall, the enhanced photocurrent in the coassembled system of DGREFEF-4T/KFKF-PDI compared to KFKF-4T/KFKF-PDI is attributed to more efficient coassembly mechanism based on complementary interactions of the peptide sequence, allowing for improved energy transfer and photocurrent generation. Comparing the level of generated photocurrents with other optoelectronically active systems (4–6), our designed biomolecular assembly should be able to deliver photocurrent-based stimulatory effects to excitable cells, such as cardiomyocytes, which is investigated here for their transient and longer-term effects in vitro.

**Fig. 4.**
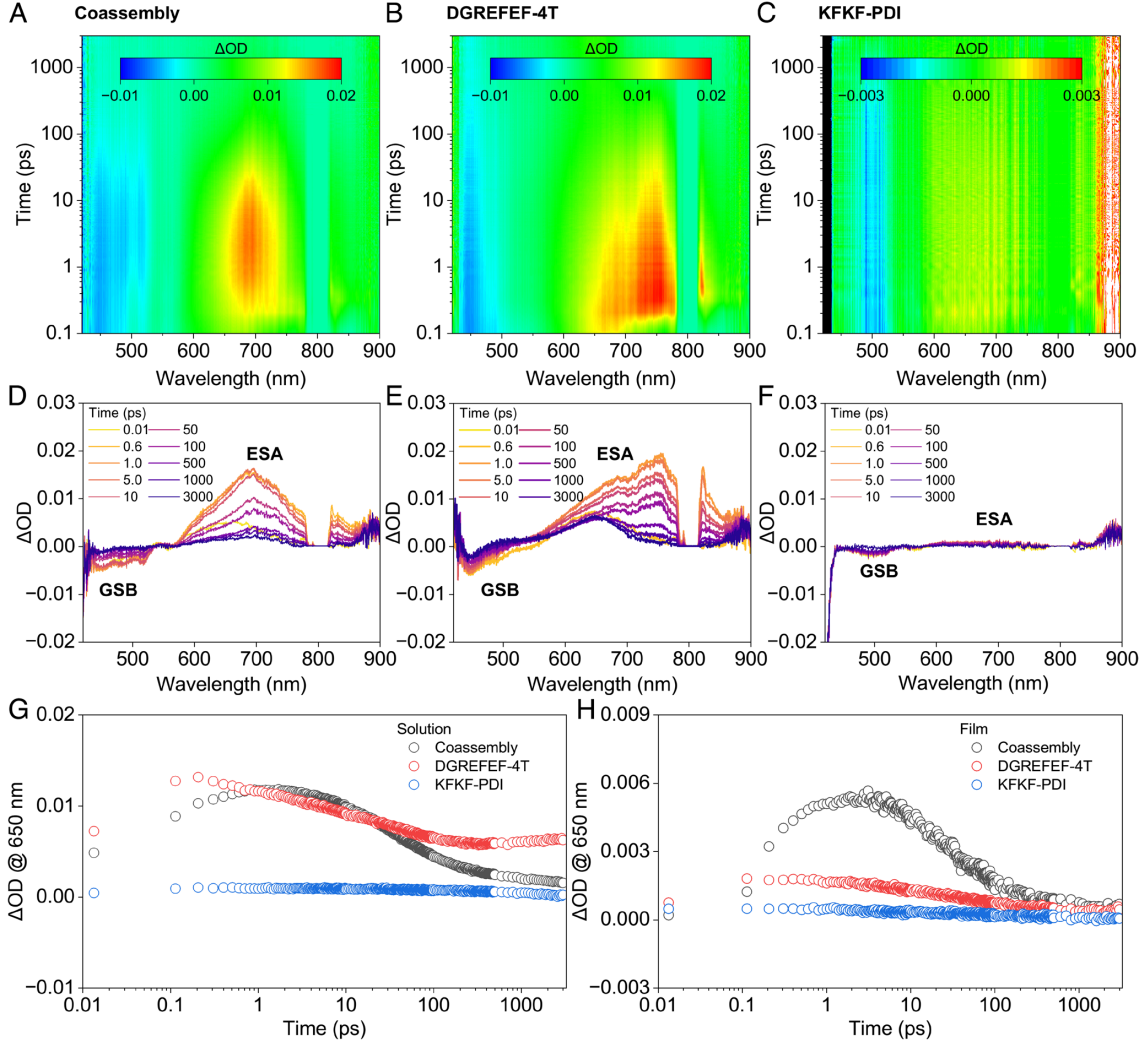
Dynamics of photoinduced excited states of peptide assemblies. Transient absorption (TA) spectra patterns of peptide assemblies in solution: (*A*) Coassembly; (*B*) DGREFEF-4T; (*C*) KFKF-PDI. TA spectra profiles of peptide assemblies in solution: (*D*) Coassembly; (*E*) DGREFEF-4T; (*F*) KFKF-PDI TA intensity decay of peptide assemblies in (*G*) solution or (*H*) film state with Tyrode’s solution. Excitation wavelength = 400 nm.

To gain insights into the time dynamics and possibility of charge transfer between the chemical species within the coassembly, we conducted transient absorption (TA) measurements on peptides in both solution and film forms (Fig. 4). In these experiments, we used 400 nm excitation for all samples aligning near the absorption maximum of the donor 4T system (*SI Appendix*, Fig. S13). The time evolution exhibits complex trends with both positive and negative transient signals, indicating ground state bleaching (GSB) and excited state absorption (ESA) at distinct spectral regions (Fig. 4 *A*–*F* and *SI Appendix*, Fig. S14). Here, we would like to focus on specific trends. Individually, DGREFEF-4T and KFKF-PDI display notably different responses. First, KFKF-PDI shows negligible signals, as the 400 nm is far away from the absorption band of this individual moiety (Fig. 4 *C* and *F*). In contrast, DGREFEF-4T demonstrates intense signals due to the excitation of photon energy in close proximity to the absorption of the molecule (Fig. 4 *B* and *E*). Such experimental arrangement allows only DGREFEF-4T to be optically excited in the coassembly system, hence, any observed changes in ultrafast dynamic would reveal the effect of carrier transfer in coassembly. For individual DGREFEF-4T, the ultrafast dynamics reveal initial decay with time constants of *t*_1_ = 16 ps (film) and *t*_1_ =21 ps (solution) upon femtosecond pulse excitation at 400 nm (*SI Appendix*, Fig. S15). The signal plateaus at 650 nm, suggesting a long-lived component with time constants exceeding *t*_2_ > 10 ns, i.e. beyond the reach of the current experimental setup (Fig. 4 *G* and *H*). However, in the coassembled form, the transient signal for DGREFEF-4T decays within *t*_2_ = 39 and 34 ps (Fig. 4 *G* and *H*), indicating a new channel for carrier loss is available for the donor in the coassembly system, attributed to its transfer from DGREFEF-4T to KFKF-PDI. To further support this conclusion, we performed time-resolved photoluminescence (TRPL) on all molecular combinations (*SI Appendix*, Figs. S16-S18). These experiments clearly show that DGREFEF-4T emission is strongly quenched by several orders of magnitude, confirming an efficient new route of carrier loss by the donor molecule through electron transfer to the PDI acceptor. In sum, these results confirm charge transfer within the coassembled system, supporting our hypothesis that peptide coassembly promotes controlled intermolecular interactions and charge transfer that supports our observed photocurrent generation behaviors.

**Fig. 5.**
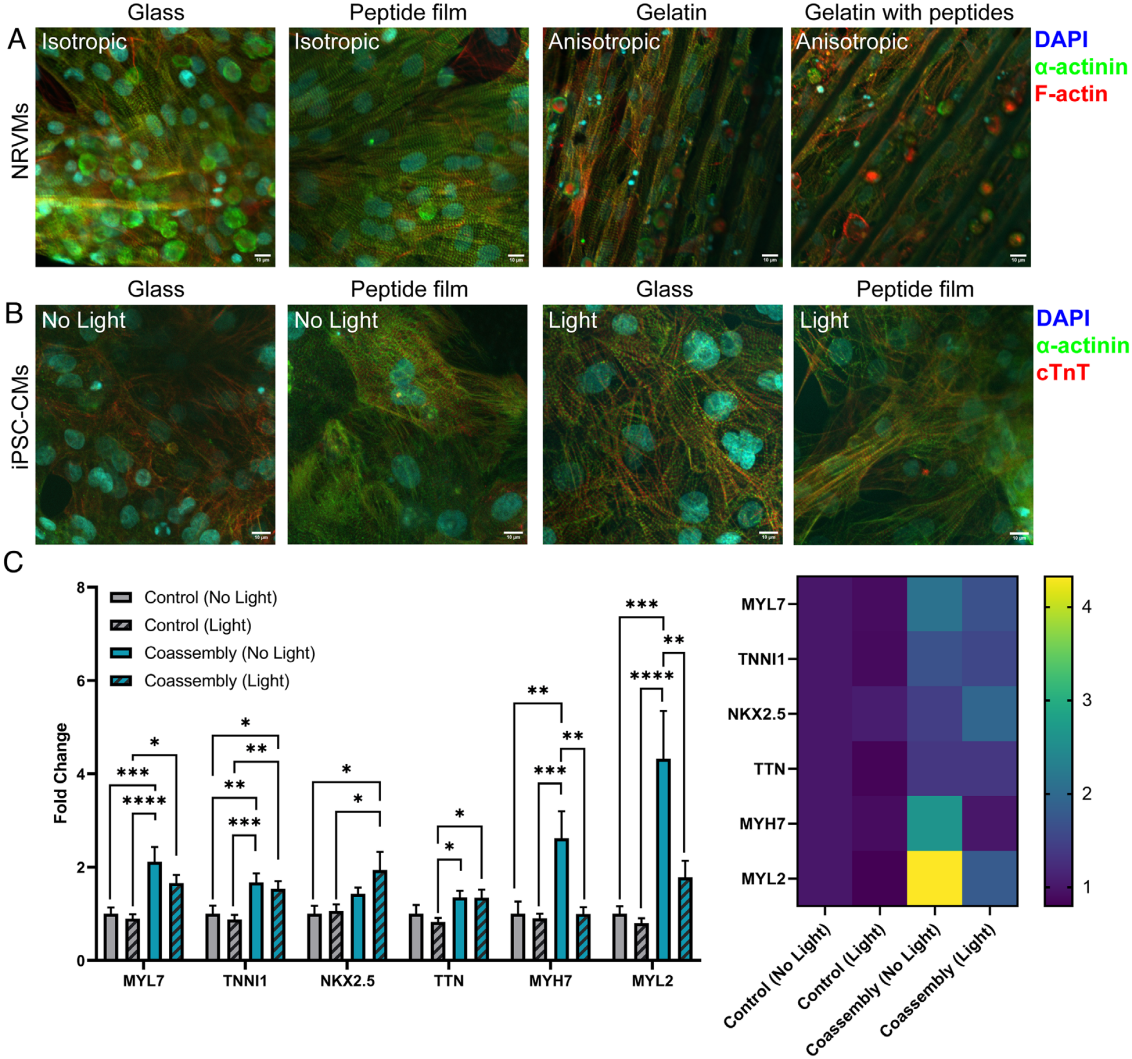
Interfacing optoelectronic peptides with cardiomyocytes. (*A*) Images of immunostained NRVMs on isotropic peptide-based films and anisotropic gelatin-peptides. (Scale bar, 10 µm.) (*B*) Images of immunostained hiPSC-CMs on peptide assemblies. (Scale bar, 10 µm.) (*C*) Gene expression profiling of hiPSC-CMs on peptide coassembly films vs. fibronectin/Geltrex-coated substrate (control) on day 22 with and without light exposure. *P*-value: *P < 0.05, **P < 0.01, ***P < 0.001, ****P < 0.0001; Statistical analysis was done using one-way ANOVA followed by Tukey’s honestly significant difference (HSD) test. n=12 to 13.

To assess the impact of optoelectronic peptide coassembly photostimulation, NRVMs and hiPSC-CMs were interfaced with the engineered peptides fabricated as biomaterial films or an interfacial layer on hydrogel scaffolds. Peptide coassembly is processed in a neutral, aqueous environment, establishing a near physiologically relevant environment for the cells. To maximize the ability of our RGD-containing biomimetic peptide coassembly films to independently promote cell adhesion, we did not use any extracellular matrix (ECM) proteins (e.g., fibronectin) to coat the surface for both NRVMs and hiPSC-CMs. The cardiomyocytes adhered well onto the peptide coassembly films and did not show any apparent issues matching the confluency of cells seeded on fibronectin/Geltrex-coated glass controls (Fig. 5 *A* and *B*). Upon photostimulation of noncontracting or arrhythmic cells using a 415 nm LED source with 2 Hz pulsing frequency, the cardiomyocytes show a change in contraction frequency across the tissue monolayer within less than 5 min of exposure to pulsed light irradiation (Movies S1–S4). This sustained photoresponsivity of cardiomyocyte contractions was not observed when photostimulation was performed without the optoelectronic peptide interface (Movies S5 and S6). In addition, as the structural alignment is an important feature for the cardiomyocytes, we tested whether the NRVMs could follow the topographical cues (pattern dimensions were 20 × 20 µm) provided by micromolding of gelatin (control; coated with fibronectin) and gelatin mixed with the peptidic coassembly (without any additional fibronectin coating) studied here. The fluorescence images of NRVMs immunostained against F-actin and *α*-actinin confirm that cells on both the patterned gelatin and patterned gelatin mixed with peptide coassembly generate anisotropic tissue line structures that follow the microgrooved topography of the substrates (Fig. 5*A* and *SI Appendix*, Fig. S19). NRVMs were able to adhere to and align on gelatin due to fibronectin coating guiding the cells. In contrast, peptide coassemblies crosslinked atop hydrogel scaffolds guided NRVMs to adhere to and follow the topographical cues of the gelatin mixed with the peptidic coassembly without any fibronectin coating. This result indicates that the peptide coassembly supports the alignment of cardiomyocytes in conjunction with the micromolded pattern of the gelatin.

Next, we assessed how the cellular morphology and gene expression of hiPSC-CMs on the peptide coassembly films were impacted by the presence of the peptidic coassembled nanostructures. The hiPSC-CMs were interfaced with the peptide coassembly films, which were then subjected to acute photostimulation at 2 Hz for 1 h per day for 3 d. Based on the fluorescence images of the immunostained cells, the cells interfaced with peptide coassemblies reveal common visual features of cardiomyocytes, such as the evident sarcomeric features based on *α*-actinin staining and expression of the marker cardiac troponin T (cTnT) (Fig. 5*B*). To further evaluate the impact of peptide interfacing and photostimulation over multiple days of treatment (vs. no stimulation and control), expressions of select cardiac markers were quantified for the hiPSC-CMs on glass vs. peptide coassembly films, with and without photostimulation treatments (Fig. 5*C*). We chose myosin light chain 7 (MYL7), troponin I1, slow skeletal type (TNNI1), and NK2 homeobox 5 (NKX2.5) as early cardiomyocytes markers and titin (TTN), *β*-cardiac myosin heavy chain 7 (MYH7), and myosin light chain (MYL2) as later cardiomyocytes markers. These genes are known to be involved in cardiac contraction and electrical conduction (73). The qPCR analysis revealed that the mere presence of the charge-complementary peptide coassemblies with optoelectronic properties generally leads to an increase in the expression of these cardiac markers, compared to when the hiPSC-CMs were seeded on fibronectin/Geltrex-coated glass substrates (control). In particular, the MYL7, TNNI1, MYH7, and MYL2 expressions for the cells on peptide coassembly films without photostimulation displayed upregulated levels of cardiac markers compared to the cells on control substrates, which demonstrate the effect of the peptide material alone. With photostimulation, upregulated MYL7, TNNI1, NKX2.5, and TTN were quantified for the hiPSC-CMs on peptidic films as compared to the control substrate, which demonstrate the effect of the peptidic material and the photocurrents that they generate during the pulsed 530 nm light exposure. Interestingly, NKX2.5, a critical regulator of cardiac development, was upregulated for the photostimulated cells on peptide coassembly films compared to the cells on peptide coassembly films without photostimulation. The upregulation of some of these select cardiac markers suggests that there are changes in cardiac muscle contraction, cardiac development, or cardiac functions at the molecular to cellular level. Future work will unveil the sensitivity of these gene expression changes to material-dependent factors such as specific sequences, choice of donor–acceptor chromophores (i.e., nature of photogenerated carriers produced), and intensity of the photocurrent generated at the cell–material interface. Given the current results and based on previous literature reports, there are a couple of material characteristics that could be attributed for the observed upregulation of select cardiac markers here. The incorporation of donor–acceptor moieties here allows for the generation of ~nA photocurrents that can facilitate localized field stimulation at the cell-peptide film interface, analogous to the conventional electric field stimulation known to aid in cardiac maturation (10, 74–77). Previous works have also reported that the inclusion of bioadhesive sequence motifs such as RGD and ECM-mimicking nanostructures can influence differentiation pathways via integrin-mediated signaling (78–82). On the other hand, we attribute the downregulation of some cardiac markers for the photostimulated peptide-interfaced cells compared to the nonphotostimulated peptide-interfaced cells to the effect of 415 nm light exposure itself (hence, separately comparing the effect of peptide interfacing under nonirradiated vs. irradiated conditions), and the potential variability in the cell populations composed of undifferentiated hiPSCs and hiPSC-CMs. Lactate purification method has been used to purify cardiomyocytes from the nonexcitable fibroblasts, which are susceptible to variations in the cell population that consists of fibroblasts and cardiomyocytes. Since the fibroblasts are not expected to be responsive to photostimulation, depending on the number of fibroblasts that were present after lactate purification, it could potentially influence the cardiac marker expression levels that we are measuring. In addition, the generation of reactive oxygen species (ROS) and the occurrence of photothermal/heating effects can happen during the photostimulation process. It is well known that excessive ROS can cause oxidative stress and could negatively affect certain functionalities of excitable cells (83, 84). Nonetheless, compared to the hiPSC-CMs on glass substrates, we demonstrate that the mere presence of the optoelectronic coassemblies is sufficient to show general upregulation of the cardiac markers investigated here. In this work, photostimulation was performed for 1 h per day on Days 18-20 at a frequency of 2 Hz. These parameters were selected based on preliminary studies; however, we acknowledge that future studies focusing on maturation mechanisms should include genotypic, structural, and functional characterizations at later differentiation timepoints and varied stimulation treatment conditions.

**Fig. 6.**
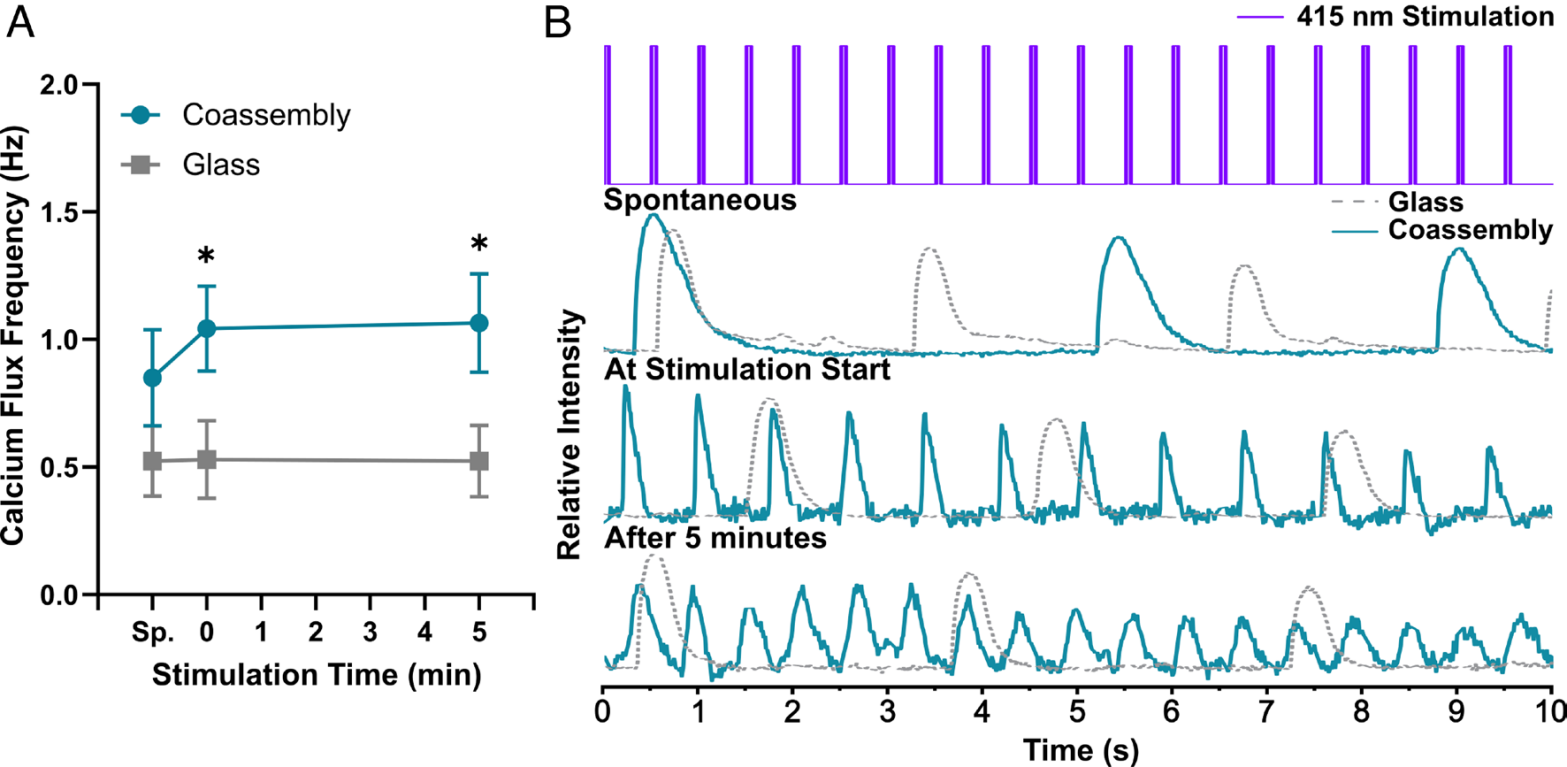
Calcium handling behavior of NRVMs on optoelectronic peptides. (*A*) Calcium flux frequency of NRVMs on glass and peptide coassembly films; *P*-value: *P < 0.05; Statistical analysis was done using the Mann–Whitney test; n=12 for control; n=10 for peptide coassembly. Sp.: Spontaneous beating. (*B*) Representative calcium transient beating curves of NRVMs measured before exposure to 415 nm pulsed LED source (spontaneous/ sp., which is no longer than 1 to 2 min before light was turned on), at the start of 415 nm light stimulation (stimulation time=0), and after 5 min of 415 nm light stimulation.

Last, to better illustrate the immediate effects of our photostimulatory peptide coassembly system on cardiac function, we evaluated the calcium handling properties of NRVMs to compare them between glass substrates coated with fibronectin (control) and those coated with peptide coassembly without fibronectin. The calcium (Ca^2+^) transients were monitored and recorded prestimulation (spontaneous), at the start of (*t* = 0), and at the end of 5 min 415 nm light stimulation (Fig. 6*A*). The NRVMs on peptide coassembly generally exhibited more rhythmic beatings compared to the NRVMs on fibronectin-coated glass. Furthermore, the calcium flux frequency for NRVMs on peptide coassembly for both conditions (at the start of photostimulation and after 5 min of photostimulation) shows statistically significant increases in beat rates compared to the cells on glass (Fig. 6*B*). NRVMs on glass (control) had no significant change in the calcium flux frequency/ beat rates in response to light exposure. Combining with the data from monitoring of contractions of cardiac monolayer interfaced with peptide films (Movies S1–S6), Fig. 6 data show that the peptide-based photostimulation causes an immediate increase in frequency of calcium transients but does not necessarily result in immediate pacing of cardiac contractions. These results suggest that stimulation due to photogeneration of charged carriers at the interface of peptidic coassemblies does not follow the same mode of action as other material interfaces reported previously for photostimulation, where a 1:1 pulse input to contractile pacing output is immediately achieved. This finding is consistent with the beat rate response of digital light processing (DLP)-printed muscular thin film actuators carrying a photocrosslinked layer of the peptidic components interfaced with cells (*SI Appendix*, Figs. S20–S26 and Movies S7 and S8). The light responsivity is otherwise not observed for non-peptide-coated monolayers or printed hydrogel actuators. Similarly, no significant increases in beat rates within the monitoring window are observed unless the constructs/samples are exposed to light. The delay in the excitation effects and their coupling with contraction may be related to the slower build-up of calcium transient amplitude needed to power calcium-dependent cardiac force generation. Additionally, while stimulation with spatial selectivity was not conducted in this work, we have previous work showing the patternability of single component π-conjugated peptides atop bioscaffolds (61). Also, the nanostructural nature of the peptide assemblies offers the opportunity for future development of site-specific photostimulation platforms via molecular recognition or nanopatterning strategies. Overall, these results show that optoelectronic peptide nanostructures are capable of transducing light stimuli into stimulatory cues for influencing cardiomyocyte contractile responses, providing the topological cues to guide the cardiomyocyte cell alignment in a directional manner, and, ultimately, influencing the gene expression profile of hiPSC-CMs. In the future, we intend to study the maturation impacts of the biomaterial and the photostimulation approach developed here at longer treatment conditions for hiPSC-CMs.

## Discussion

In summary, we developed a biomolecular electronic platform for cardiac photostimulation using π-conjugated peptide nanostructures with engineered donor–acceptor interactions via charge-complementary coassembly. Here, we demonstrate that sequence design influences charge and energy transport within nanoassemblies of π-conjugated peptides and their interactions with excitable cardiomyocytes. We integrated a p-type and an n-type organic semiconductor with charge-complementary peptide sequences to control the assembly structures and to create photocurrent-generating cardiac biointerfaces out of the coassembled peptide nanostructures. Importantly, the observed sequence pair-driven photocurrent generation within our peptide nanostructures exhibits their potential for use in bioelectronic applications. The peptide- and light-induced stimulation of cardiomyocytes through the topographical/phenotypic/genotypic influence of our engineered biomolecular coassemblies on hiPSC-CMs represents a strategy for manipulating cell behavior without the need for prior genetic modification of the cells of interest. The specific mechanistic role of cues or physical characteristics presented by the biomaterial, as well as dynamics of the photocurrent generation mechanism as it relates to the timescale of cardiac excitation–contraction coupling, will be the subject of future work. Unlike traditional electrical stimulation approaches that operate in a bulk manner, the biomolecular nature of these optoelectronic peptide coassemblies with substrate patternability and sequence-based tunability of molecular and nanoscale organization may enable localized interactions with cells through sequence design, offering the potential for enhanced spatial resolution and specificity in future applications. Beyond comparing the effect of sequence complementarity versus sequence matching on photocurrent outputs, future work on a larger library of peptide sequences could also enable molecular tunability toward a broader range of photocurrents, targeting the needs of different cell types. For a broader utility of this biomolecular electronic platform for cardiac photostimulation in the future, we note that the long-term stability of these peptide materials needs further testing in ex vivo tissue environments or animal models, beyond the timescale shown for the stability experiments in *SI Appendix*, Fig. S12. Moreover, while the photostimulation parameters maintained the structural phenotype expected for NRVMs and hiPSC-CMs, light penetration in thicker or in vivo cardiac tissues requires further optimization of the material design and stimulation treatment methods. Deeper light penetration could be addressed by selecting donor–acceptor π-systems that operate in the far-red or near-infrared regions. As for the manufacturability and processability of the biomaterial system here, future work will take advantage of automation strategies to scale up the synthesis of π-conjugated peptides and fabrication of the associated devices/substrates. Here, we already show that the molecular versatility of the biomolecular coassembly used enables the synthesis of photocrosslinkable versions compatible with light-based 3D-printing methods, helpful in fabricating 3D cardiac actuator constructs (*SI Appendix*, Fig. S25).

Overall, the findings of this work clearly show that the designed biomolecular interface can successfully influence cardiac structure and function by modulating the molecular features at the biomaterial surface, advancing the strategies available for biomaterial-aided cardiac tissue engineering.

## Materials and Methods

Solid phase peptide synthesis and on-resin coupling techniques were used to generate the materials reported here. Structural and spectral characterizations of the materials were performed using techniques such as TEM, solid-state NMR spectroscopy, GISAXS, CD spectroscopy, and steady-state/time-resolved absorption and photoluminescence spectroscopy. Photocurrent generation was measured in both solution and film states at neutral pH. Substrates crosslinked with the peptide-based donor–acceptor coassembly system were interfaced with NRVMs and hiPSC-CMs to investigate the influence of the material and light exposure on the gene expression profile, structural features, and functional features of these in vitro cardiac cultures. Statistical analyses were performed using one-way ANOVA for qPCR and the built-in Mann–Whitney U test for calcium handling. Detailed experimental procedures, information on materials used, and supplemental figures are provided in the *SI Appendix*.

## Supplementary Material

Appendix 01 (PDF)

Movie S1. Video of NRVMs interfaced with coassembled KFKF-PDI/DGREFEF-4T on PEDOT:PSS film before light stimulation.

Movie S2. Video of NRVMs interfaced with coassembled KFKF-PDI/DGREFEF-4T on PEDOT:PSS film during light stimulation of 415 nm with 2 Hz pulsing frequency.

Movie S3. Video of NRVMs interfaced with coassembled KFKF-PDI/DGREFEF-4T on PEDOT:PSS film before light stimulation.

Movie S4. Video of NRVMs interfaced with coassembled KFKF-PDI/DGREFEF-4T on PEDOT:PSS film during light stimulation of 415 nm with 2 Hz pulsing frequency.

Movie S5. Video of NRVMs on glass control substrate before light stimulation.

Movie S6. Video of NRVMs on glass control substrate during light stimulation of 415 nm with 2 Hz pulsing frequency.

Movie S7. Video of NRVMs on a DLP-printed cantilever before and during (t=17 min) light stimulation of 415 nm with 2 Hz pulsing frequency.

Movie S8. Video of NRVMs on a DLP-printed cantilever after 2 min of light stimulation of 415 nm with 2 Hz pulsing frequency, showing an actuating cardiac biohybrid construct that bears an interfacial layer of photocurrent-generating peptides.

## Data Availability

All study data are included in the article and/or supporting information.
