# Supplementary material for: Complementary biomolecular coassemblies direct energy transport for cardiac photostimulators
Source: Proc Natl Acad Sci U S A. 2025 Sep 4;122(36):e2509467122. doi: 10.1073/pnas.2509467122 (PMC12435254; doi:10.1073/pnas.2509467122)
Supplement: Supplementary file 1 — Appendix 01 (PDF) [file pnas.2509467122.sapp.pdf]

Supporting Information for
Complementary biomolecular coassemblies direct energy transport for cardiac photostimulators

Ze-Fan Yao,[a,b,1] Sujeung Lim,[a,1] Yuyao Kuang,[a] Emil M. Lundqvist,[c] Natalie Celt,[c] Caleb O. Chung,[a] Kathryn K. Lee,[a] Krystal Nguyen,[a] Lanie Le,[c] Sheng Wei Tang,[b] Griffin M. Milligan,[b] Phillip Kohl,[d] Tarunya Rao Sudarshan,[e] Youli Li,[d] Asuka Eguchi,[f,g] Anant K. Paravastu,[e,h] Michael V. Zaragoza,[g,i] Dmitry A. Fishman,[b] Herdeline Ann M. Ardoña[a,b,c,g]*

[a]Department of Chemical and Biomolecular Engineering, Samueli School of Engineering, University of California, Irvine, CA 92697, USA
[b]Department of Chemistry, School of Physical Sciences, University of California, Irvine, CA 92697, USA
[c]Department of Biomedical Engineering, Samueli School of Engineering, University of California, Irvine, CA 92697, USA
[d]Materials Research Laboratory and BioPACIFIC MIP, University of California, Santa Barbara, CA 93106, USA
[e]School of Chemical and Biomolecular Engineering, Georgia Institute of Technology, Atlanta, GA 30332, USA
[f]Department of Physiology & Biophysics, School of Medicine, University of California, Irvine, CA 92617, USA
[g]Sue & Bill Gross Stem Cell Research Center, University of California, Irvine, CA 92697, USA
[h]Parker H. Petit Institute for Bioengineering and Biosciences, Georgia Institute of Technology, 315 Ferst Drive, Atlanta, 30332, GA, USA
[i]Departments of Pediatrics and Biological Chemistry, School of Medicine, Irvine, CA 92617, USA

[1]Ze-Fan Yao and Sujeung Lim contributed equally to this work.

*Herdeline Ann M. Ardoña
Email: hardona@uci.edu

This PDF file includes:

Supporting text
Figures S1 to S26
Legends for Movies S1 to S8
SI References

Other supporting materials for this manuscript include the following:

Movies S1 to S8

Supporting Information Text

1. Materials

Wang resins loaded with Asp(OtBu) and Lys(Boc), 9-fluorenylmethoxycarbonyl (Fmoc) protected amino acids were obtained from Advanced ChemTech. *O*-(benzotriazol-1-yl)-*N*,*N*,*N*′,*N*′-tetramethyluroniumhexafluorophosphate (HBTU), ethyl (hydroxyimino)cyanoacetate (Oxyma), *N,N′*-diisopropylcarbodiimide (DIC), triisopropylsilane (TIPS), trifluoroacetic acid (TFA), *N*-methyl pyrrolidone (NMP), and benzotriazol-1-yl-oxytripyrrolidinophosphonium hexafluorophosphate (PyBOP) were obtained from Oakwood Chemicals. Acetonitrile, methanol, formic acid, ammonium hydroxide, and dichloromethane (DCM) were obtained from Fisher Scientific. *N*,*N*-dimethyl formamide (DMF), diisopropylethylamine (DIPEA), piperidine, 5,5'-bis-tributylstannyl-[2,2']-bithiophene, perylene-3,4,9,10-tetracarboxylic dianhydride (PTCDA), poly(2,3-dihydrothieno-1,4-dioxin)-poly(styrenesulfonate) (PEDOT:PSS), (3-glycidyloxypropyl)trimethoxysilane (GOPS), (3-aminopropyl)triethoxysilane (APTES), and *N*-hydroxysuccinimidewere (NHS) were purchased from Sigma-Aldrich. 1-ethyl-3-(3-dimethylaminopropyl)carbodiimide (EDC) was obtained from AA Blocks Inc. Finally, [2,2′:5′,2′′:5′′,2′′′-quaterthiophene]-5,5′′′-dicarboxylic acid was prepared according to the literature.(1) All commercially available chemicals were used without further purification unless otherwise noted. Solvents were degassed by sparging with nitrogen for 30 to 90 min while drying over 4 Å molecular sieves before use.

2. Peptide synthesis

The synthetic procedures for π-conjugated peptides were conducted according to the reported methods.(2–4) The peptide segments of these supramolecular units were synthesized using a Liberty Blue peptide synthesizer, starting with a Wang resin preloaded with the first amino acid (Wang-Asp(OtBu) = 0.6 mmol g^{-1}, for DGREFEF and DGREFEFKa, and Wang-Lys(Boc)=0.53 mmol g^{-1}, for KFKF and KFKFKa) sequentially coupled to Fmoc-protected amino acids together with Oxyma and DIC as activators. In general, the final Fmoc deprotection was carried out by treating the resins with a 20% piperidine solution in DMF for 2 min, followed by filtering, adding piperidine solution and mixing for 10 min. Then, the resins were filtered and washed 3× with NMP, methanol and DCM. Following completion of the oligopeptide sequence and deprotection of the last amino acid residue, the resins were treated either with 2,2′:5′,2′′:5′′,2′′′-quaterthiophene]-5,5′′′-dicarboxylic acid dissolved in NMP at room temperature for 48 h or with PTCDA (0.5 eq.) dissolved in pyridine at 135 °C for 72 h, and both were activated by PyBOP (3 eq.) and DIPEA (10 eq.). After coupling, the resins were filtered, washed 3× with NMP, methanol and DCM. Then, the resins were returned to the peptide chambers and again were subjected to a standard wash cycle: 3× NMP, 3× MeOH, and 3× DCM. The resins were treated with a cleavage cocktail consists of 9.5 mL TFA, 250 µL TIPS, and 250 µL Milli-Q water for 2–3 h. The peptide solution was filtered from the resin beads, washed 3× with DCM, and was concentrated by evaporation under reduced pressure. The crude peptides were then precipitated from the solution with 90 mL of cold diethyl ether and isolated through centrifugation. The resulting pellet was triturated with diethyl ether to yield crude products, which were dissolved in Milli-Q water and ammonium hydroxide for DGREFEF-4T and DGREFEFKa-4T and in Milli-Q water for KFKF-PDI and KFKFKa-PDI before freeze-drying to obtain solid products. The purification was performed to purify the crude peptide products using Agilent 1260 Infinity II Preparative HPLC system (column: Zorbax Eclipse XDB-C8, 21.2 × 250 mm). The mobile phase used consists of a 0.1% ammonium formate in Milli-Q water (pH 8–9; for DGREFEF-4T and DGREFEFKa-4T) and acetonitrile or 0.1% formic acid in Milli-Q water (pH 2–3; for KFKF-PDI and KFKFKa-PDI) and acetonitrile. The purity for DGREFEF-4T and KFKF-PDI were confirmed using analytical HPLC (column: Zorbax 300SB-C3, 4.6 × 150 mm) using the same solvent compositions as the preparative HPLC system. Matrix-assisted laser desorption/ionization (MALDI) (AB Sciex 5800) was performed to confirm the mass of DGREFEF-4T and KFKF-PDI. The matrix used was α-cyano-4-hydroxycinnamic acid (CHCA). The purity was monitored for DGREFEFKa-4T and KFKFKa-PDI using Waters Acquity ESI-UPLC-QDA (Electrospray Ionization-Ultra Performance Liquid Chromatography-Single Quadrupole Mass Detector) with reversed phase C4 column (Waters Acquity, 50 mm C4). Samples were prepared by dissolving the KFKFKa-PDI and DGREFEFKa-4T peptides in 0.1% formic acid in Milli-Q water and 0.1% ammonium formate in Milli-Q water, respectively. Chromatographs and mass spectra were obtained in positive and negative modes. ^{1}H-NMR spectra were obtained using a Bruker

Avance 500 MHz. Chemical shifts are reported in parts per million (ppm) relative to residual solvent in D_2O of 4.79. DGREFEF-4T: ^{1}H- NMR (500 MHz, D_2O) δ, ppm: 7.07-7.25 (thiophene-H, 8H, Phe-aromatic H, 16H), 6.25-6.42 (Phe-aromatic H, 4H), 4.78 (Asp-αH, 2H, Phe-αH, 4H), 4.26-4.54 (Arg-αH, 4H, Glu-αH, 8H), 3.89-4.02 (Gly- αH, 4H), 2.90-3.06 (Arg-δH, 4H, Phe-βH, 8H), 2.54-2.68 (Asp-βH, 4H), 1.83-2.17 (Glu-γH, 8H, Glu-βH, 8H), 1.56-1.69 (Arg-βH, 4H, Arg-γH, 4H). KFKF-PDI: ^{1}H-NMR (500 MHz, D_2O) δ, ppm: 7.04-8.18 (PDI-H, 8H, Phe-aromatic H, 20H), 4.01-4.68 (Lys-αH, 4H, Phe-αH, 4H), 2.93-3.27 (Phe-βH, 8H), 2.51-2.86 (Lys-εH, 8H), 1.34-1.65 (Lys-δH, 8H; Lys-βH,8H; Lys-γH, 8H). The structural characterizations KFKF-4T were previously reported in Lundqvist et al.(4)

3. Molecular simulations

Geometries of single molecule peptide units were first optimized using GFN2-xTB method.(5) Electrostatic potential (ESP) maps were generated according to a density-functional theory (DFT) calculation at the level of B3LYP-D3/def2-SVP. Then, two molecules were manually stacked as the aggregated structures and further optimized with GFN2-xtb method. The visualization and analysis of simulated structures were performed using Multiwfn and Mercury.(6, 7) The computational work described above utilized the infrastructure for high-performance and high-throughput computing, research data storage and analysis, and scientific software tool integration built, operated, and updated by the Research Cyberinfrastructure Center (RCIC) at the UC Irvine. The RCIC provides cluster-based systems, application software, and scalable storage to directly support the UCI research community (Website: https://rcic.uci.edu).

4. Peptide assembly and film fabrication

PEDOT:PSS (1.3 wt%) was mixed with 1 v/v% GOPS and 1 v/v% APTES. The glass substrates with or without patterned gold electrodes were cleaned by O_2 plasma for 10 min and then the PEDOT:PSS layer was deposited on glass substrates by spin-coating at 3000 rpm for 60 s. For deposition of coassembly and KFKF-PDI, the substrates were further treated with 0.5% glutaraldehyde solutions for 1 h. The π-conjugated peptides (DGREFEF-4T and KFKF-PDI) were directly dissolved in the UltraPure distilled water with a concentration of 2 mM. DGREFEF-4T and KFKF-PDI solutions were mixed with the same volume to give the coassembly with a fixed total concentration of 2 mM. The peptide solutions of coassembly or DGREFEF-4T were mixed with crosslinkers of 1% EDC/NHS solutions and then dropped onto the fabricated PEDOT:PSS surface. The peptide solutions of KFKF-PDI were directly dropped onto the fabricated PEDOT:PSS surface. All the samples with the dropped solution were left in the closed petri dish overnight. The cover of the petri dish was opened after one night for solvent evaporation. After natural drying, the samples were washed with phosphate buffered saline (PBS) (1X) and UltraPure water each three times and dried overnight for further use.

5. Spectral characterizations

Linear absorption and photoluminescence

All solution samples for absorption and photoluminescence (PL) spectra were prepared with a concentration of 33 μM by dissolving lyophilized peptides in Milli-Q water. All spectra were measured using a 1 cm quartz cuvette at room temperature. Absorption spectra were collected using Agilent Cary 100 UV-vis spectrophotometer and PL data were collected using Cary Eclipse fluorescence spectrometer with excitation wavelength of 400 nm. Circular dichroism (CD) spectra were acquired using Jasco J-810 circular dichroism spectropolarimeter.

Ultrafast transient absorption spectroscopy

Transient absorption (TA) spectra were collected using a custom-built ultrafast setup in a conventional pump-probe configuration, employing a femtosecond amplified laser system (Spitfire Ace, Spectra-Physics/MKS Instruments). A small portion of the fundamental 800 nm pulse (80 fs, 100 mW, 1 kHz) was partially focused onto a sapphire plate to generate a white-light continuum, serving as the broadband probe pulse. Another portion of the amplified output was frequency-doubled to produce a 400 nm pulse (80 fs, 0.5 mW), which acted as the pump beam. The pump and probe beams were overlapped on the sample, placed in a 1 mm path-length quartz cuvette. A delay line (Newport, MKS Instruments) controlled the time delay between the pump and probe

pulses, while a CCD-equipped spectrometer (Oriel, Newport, MKS Instruments) recorded the transient signal for each spectral component. All measurements were conducted within the linear response regime of the detection system. Solution and film samples used here to obtain measurement were prepared the same way as described in Section 4-*Peptide assembly and film fabrication*, and in Tyrode's solution (119 mM NaCl, 5 mM KCl, 25 mM HEPES pH 7.4, 2 mM $CaCl_2$, 2 mM $MgCl_2$, 6 g/L glucose in 500 mL MilliQ water).

Ultrafast time-resolved photoluminescence
Time-resolved photoluminescence (TRPL) measurements were performed via time-correlated single-photon counting, using a femtosecond oscillator (800 nm, 80 MHz, Mai Tai, Spectra-Physics, MKS Instruments) with its second harmonic (400 nm, 3 mW) as the excitation source. Emitted photoluminescence was collected and directed to a spectrometer (Oriel, Newport, MKS Instruments) equipped with a fast photomultiplier tube (Hamamatsu). Solution and film samples used here to obtain measurement were prepared the same way as described in Section 4-*Peptide assembly and film fabrication*, and in Tyrode's solution (119 mM NaCl, 5 mM KCl, 25 mM HEPES pH 7.4, 2 mM $CaCl_2$, 2 mM $MgCl_2$, 6 g/L glucose in 500 mL MilliQ water).

Solid-state nuclear magnetic resonance (NMR) experiments
Solid-state NMR experiments were performed using a 500 MHz Bruker spectrometer equipped with a 3.2 mm HCN MAS (magic angle spinning) probe and a 3.2 mm Bruker Low-E $^{1}H/^{13}C/^{15}N$ NMR probe. Calibration was conducted by measuring the NMR signal of adamantane, confirming its chemical shifts relative to TMS at 38.38 ppm and 29.45 ppm.(8) 1D ^{13}C cross polarization magic angle spinning (CPMAS) experiments were carried out at an MAS frequency of 10 kHz, with signal averaging over a 24 h scanning period.(9, 10)

6. Morphological measurements

Transmission electron microscopy (TEM)
2 mM solutions of individual peptides or coassemblies were dropped onto 200 mesh copper grids with carbon coating. The grids were washed with DI water and then stained with a 2% uranyl acetate solution. The grids were allowed to dry prior to TEM imaging. C_s-Corrected TEM was performed using a JEOL JEM-ARM300F Grand Arm in IMRI operated at an accelerating voltage of 300 kV. All TEM images were analyzed and visualized using Gatan Digital Micrograph software.

Grazing incidence small-angle X-ray scattering (GISAXS)
GISAXS was performed using the BioPACIFIC MIP (Materials Innovation Platform) SAXS instrument at University of California, Santa Barbara. The SAXS instrument was custom-built using a high brightness liquid metal jet X-ray source (Excillum MetalJet D2+ 70 keV) and a 4-megapixel hybrid photon-counting X-ray area detector (Dectris Eiger2 R 4M). GISAXS data analysis was performed using the Irena software package.(11)

High-magnification optical microscopy and confocal microscopy
The assembly of DGREFEF-4T/KFKF-PDI and KFKF-4T/KFKF-PDI solutions was evaluated using an Olympus BX43F optical microscope (Olympus, Tokyo, Japan) in transmission mode at 50× and 100× magnification for brightfield images and Olympus Fluoview FV300 confocal microscope for fluorescence images. 33 μM solutions of the co-assembled materials were dropcasted onto a microscope slide, mounted using a small coverslip, and then sealed to ensure the solution would not dry out. Samples were imaged within 24 h of preparation. Brightfield images and fluorescence images were processed using ImageJ.

7. Device fabrication and photocurrent measurement

After the deposition of π-conjugated peptides on PEDOT:PSS/glass substrates with patterned gold electrodes, time-dependent current profiles were measured to assess the photocurrent generation. For photocurrent generation measurements, the fabricated devices were illuminated by a 415-nm or 530-nm fiber-coupled light-emitting diode (LED) light source that is controlled by a programmable pulse generator. Device measurements were performed using a probe station equipped with a

Keithley 4200 SCS semiconductor parameter analyzer. The incident light pulse was generated by a Prizmatix (FC-LED, 415 nm, 16 mW cm^{-2} or 530 nm, 12 mW cm^{-2}) fiber-coupled LED light source programmed to the pulse frequency of 1 or 2 Hz and exposure time of 50 ms.

8. Neonatal rat ventricular myocytes (NRVMs) cell culture

NRVMs used for this work were obtained from two-day old Sprague Dawley neonatal rats (mixed male and female neonates, Charles River®). The NRVM isolation protocol has been approved by the UCI IACUC Committee, under approval number AUP-23-089. After isolation from the rat pups, ventricular cardiomyocytes were incubated over a 14 h period in trypsin (1 mg/mL) to dissolve connective tissue. The tissue was enzymatically dissociated using collagenase (1 mg/mL) and mechanically triturated by gentle vortexing in a bead bath at 75 rpm as well as by pipetting with a serological pipette. The resulting cell suspension was collected, leaving behind any remaining tissue, and the digestion process was repeated until complete tissue dissociation was achieved. To enrich the population of cardiomyocytes, the dissociated cells were plated onto T-75 and T-175 flasks for 45 min each, allowing adherent cardiac fibroblasts to attach while non-adherent cardiomyocytes remained in suspension. Cells were seeded at a concentration of 526,000 cell/cm^2 for optimal confluency on peptide films on PEDOT:PSS or line-patterned gelatin mixed with peptides in 24-well plates. NRVMs were maintained using M199 medium supplemented with glucose, L-glutamine, vitamin B_{12}, HEPES, MEM-α, and 30% FBS on day 1, the same media supplemented with 10% FBS on day 2 and the same media supplemented with 2% FBS on day 4. Cell culture medium was replaced every 2 days until day 5. Cells were stimulated using the 415-nm LED light source (16 mW cm^{-2}) programmed to the pulse frequency of 2 Hz and exposure time of 50 ms.

9. Human induced pluripotent stem cell-derived cardiomyocytes (hiPSC-CMs) cell culture

The hiPSCs used in this work were generated by culturing and reprogramming Lonza Fibroblast from a 51-year-old male (NHDF-Ad-Human Dermal Fibroblasts, Adult, catalog # CC-2511; Lot # 0000293971). This line was karyotyped to have normal chromosome constitution, and pluripotency was checked by immunocytochemistry (ICC).(12) The hiPSCs were cultured on Geltrex™ (Gibco, catalog # A1413302)-coated 6-well plates (Corning, catalog # 353046) in mTeSR™1 media (Stemcell Technologies, catalog # 85850) with daily media changes. Cells were passaged at 70-80% confluency at 1:6 by following these steps: washing cells once with 2 mL/well of dPBS (1X) (without calcium and magnesium; Gibco, catalog #14190250). 1 mL/well ReLeSR™ (Stemcell Technologies, catalog # 100-0483) was added and aspirated to remove ReLeSR™ within 1 min and the colonies were exposed to only the residual liquid for 4 more min. After 4 min, the plate was gently tapped to lift off most of the colonies from the plate. 1 mL/well of mTeSR™1 media was added to transfer the detached cell aggregates to a 15 mL tube using a 5 mL serological pipette. Cell aggregates were mixed up and down using a 5 mL serological pipette before plating them into a coated 6-well plate. After two passages of iPSCs, when iPSCs reach 70-80% confluency, they were singularized to set up for the CM differentiation. In brief, cells were washed with 2 mL/well of dPBS (1X) (without calcium and magnesium), and 1 mL/well of TrypLE™ (Gibco, catalog # 12563011) was added to each well. The plate was placed in the incubator at 37°C for 10 min. 1 mL of mTeSR™1 with Y-27632 (1:1000) (Cellagen Tech, catalog # C9127-2s) was added to each well and they were gently pipetted up and down once and transferred to a 15 mL tube using a 10 mL serological pipette. The cell suspension was centrifuged at 1000 rpm for 5 min and the suspension was aspirated, leaving only the cell pellet inside the tube. The pellet was gently mixed, and cells were counted using Countess™ 3 Automated Cell Counter (Invitrogen, catalog# AMQAX2000). Cells were seeded with the optimized density per well and the plates were placed inside the incubator. The media was changed using mTeSR™1 media for 2 more days until day 0 (D0) of differentiation. The GiWi cardiac differentiation protocol was used to produce CMs.(13, 14) On days 12 and 14, hiPSC-CMs were purified using the lactate media, made with RPMI-1640 no glucose+B27^{+}+5 mM lactate (Sigma-Aldrich, catalog # L7900-100). On day 14, glass substrates were coated with the mixture of Geltrex™ (Gibco, catalog # A1413302) and fibronectin (Sigma-Aldrich, catalog# F0895-2MG) and peptide films on PEDOT:PSS substrates without any ECM coating were prepared in 24-well plates (Corning, catalog # 353047) a sterile condition. On day 15 of differentiation, hiPSC-CMs were washed with dPBS (1X) (without calcium and magnesium) twice

and dissociated using STEMdiff™ Cardiomyocyte Dissociation Medium (Stemcell Technologies, catalog # 05026) following the manufacturer's protocol. Cells were quenched using STEMdiff™ Cardiomyocyte Support Medium and centrifuged at 1200 rpm for 5 min. The cell suspension was aspirated and resuspended in the same media. Cells were counted using Countess™ 3 Automated Cell Counter (Invitrogen, catalog# AMQAX2000) and seeded at 789,000 cells/cm^2 on peptide films on PEDOT:PSS and glass as a control in 24-well plates as a monolayer. On the next day, the medium was changed back to RPMI-1640+$B27^+$ media. The cells were stimulated on days 18-20 for 1 h daily at 2 Hz, using 530 nm LED light source (Prizmatix Ltd; using the longer wavelength source at 12 mW cm^{-2} for prolonged stimulation regimens).

10. RNA isolation, cDNA conversion and qPCR

The hiPSC-CMs on glass and on peptide coassembly substrates were lysed using 500 μL Trizol (Invitrogen, catalog # 15596026). 100 μL chloroform was added to each sample and each sample was shaken for 3 min. The samples were centrifuged for 15 min at 12000 rpm at 4 °C. The aqueous phase containing the RNA was transferred to new tubes. 200 μL of 70% ethanol was added to the samples and were mixed. A PureLink™ RNA Mini Kit (Invitrogen, catalog # 12183018A) was used to purify the RNA following the manufacturer's protocol. The final RNA concentration was measured and stored at −80 °C for further processing. RNAs were converted to complementary DNA (cDNA) using High-Capacity cDNA Reverse Transcription Kit (Applied Biosystem, catalog # 4368814) according to the manufacturer's protocol. Quantitative polymerase chain reaction (qPCR) was done using QuantStudio3 (Thermo Fisher). The reaction was performed by following these conditions: polymerase activation at 95 °C for 20 s followed by 50 cycles of the thermal cycling of 95 °C for 1 s and 60 °C for 20 s. Based on the manufacturer's protocol, each reaction mixture contains TaqMan™ Fast Advanced Master Mix for qPCR (Applied Biosystems, catalog # 4444963) and the following Taq™ Gene Expression Assay (FAM) (Applied Biosystems, catalog # 4331182): myosin light chain 7 (MYL7), troponin I1, slow skeletal type (TNNI1), NK2 homeobox 5 (NKX2-5), titin (TTN), β-cardiac myosin heavy chain 7 (MYH7), myosin light chain (MYL2), glyceraldehyde-3-phosphate dehydrogenase (GAPDH). GAPDH was used as the endogenous control gene. Each PCR reaction was performed in triplicate. The data was processed using Prism-GraphPad (version 9.5.1). Statistical analysis was performed using in-built one-way ANOVA (Analysis of Variance).

11. NRVMs and hiPSC-CMs immunostaining

Both cell types were fixed using 4% paraformaldehyde for 10 min, then permeabilized with 0.1% Triton X-100 for 10 min. 5% BSA was used to incubate the cells for 30 min at room temperature. NRVMs were incubated with appropriate primary or secondary antibodies in 1% bovine serum albumin and PBS (1X) to fluorescently label actin, nuclei, and *α*-actinin for 2 h. Monoclonal anti-*α*-actinin (sarcomeric) antibody produced in mouse (Sigma Aldrich, Merck KGaA, clone EA-53, ascites fluid, catalog # A7811) and goat anti-mouse IgG (H+L) cross-adsorbed secondary antibody, Alexa Fluor™ 488 (Invitrogen by Thermo Fisher Scientific Inc., catalog # A-11001) were used to stain *α*-actinin. Alexa Fluor™ 546 Phalloidin was used to stain actin. The hiPSC-CMs were incubated with appropriate primary or secondary antibodies in 1% bovine serum albumin and PBS (1X) to fluorescently label cardiac troponin T (cTnT), nuclei, and *α*-actinin for 2 h. Alpha actinin 2 recombinant rabbit monoclonal antibody (7H1L69) (Invitrogen, catalog # 701914) and goat anti-rabbit IgG (H+L) cross-adsorbed secondary antibody, Alexa Fluor™ 488 (Invitrogen, catalog # A-11001) were used to stain *α*-actinin. Cardiac troponin T monoclonal antibody (13-11) (Thermo Fisher Scientific, catalog # MA5-12960) and goat anti-mouse IgG (H+L) highly cross-adsorbed secondary antibody, Alexa Fluor™ 546 (Invitrogen, catalog # A-11030) was used to stain cTnT. For both cell types, and 4′,6-diamidino-2-phenylindole (DAPI) was used to stain nuclei. After washing three times with 0.5% BSA and PBS (1X), substrates were mounted onto glass slides and imaged using Keyence BZ-X800 inverted fluorescence microscope (Keyence®, Osaka, Japan) and Olympus Fluoview FV300 confocal fluorescence microscope. Cell images were processed with BZ-X800 Analyzer and ImageJ.

12. Calcium Handling

NRVMs were seeded on glass coated with fibronectin and on peptide coassembly without fibronectin at a seeding density of 789,000 cells/cm^2. NRVMs were maintained using M199 medium supplemented with glucose, L-glutamine, vitamin B_{12}, HEPES, MEM-α, and 30% FBS on day 1, the same media supplemented with 10% FBS on day 2 and the same media supplemented with 2% FBS on day 4. On day 4, the substrates with NRVMs were checked under the microscope to confirm the cell health, cell adhesion, tissue confluency and substrate stability to control the quality of the samples. The substrates that met the selection criteria were chosen to be further tested for calcium flux. X-Rhod-1 (Invitrogen by Thermo Fisher Scientific Inc., catalog # X14210) was prepared at a stock concentration of 1 µg/mL using sterile DMSO. Calcium flux was visualized by incubating the NRVM constructs with X-Rhod-1 (Invitrogen by Thermo Fisher Scientific Inc., catalog# X14210) at a final concentration of 5 µM for 30 min at 37°C. After incubation, the cells were washed one time using warm 1X PBS and then placed in a Zeiss Axio Observer inverted microscope (Carl Zeiss, Oberkochen, Germany) and a Photometrics PRIME 95B SCMOS camera for imaging. The temperature and CO_2 concentration for the cell environment was maintained through a CO_2 Module S1 (Part No: 810-450001, company: PeCon GmBH, headquarters: Erbach, Germany) and Temp Module S1 (Part No: 800-450531, company: PeCon GmBH, headquarters: Erbach, Germany) and Heating Unit XL S2 (Part No: 840-450562, company: PeCon GmBH, headquarters: Erbach, Germany). The light stimulation apparatus used were the same as described in “Device fabrication and photocurrent measurement” and was set up to allow for the light (415-nm) to stimulate the cardiomyocytes while simultaneously recording the calcium signal. The calcium flux was then recorded on the microscope before light stimulation, at the start of light stimulation and after 5 min of light stimulation. Videos of the calcium signals were then analyzed using ImageJ (NIH) by looking at the changes in intensity through the video. Calcium flux peaks were counted manually in each video, then divided by the total time. To better visualize the peaks, the videos were processed using a custom ImageJ macro to remove overlapping signals caused by the photostimulation. The sample size for control was n= 12 and for peptide coassembly was n=10, in which these samples were from multiple batches of harvests and from multiple wells. The data was plotted using Prism-GraphPad (version 9.5.1). Statistical analysis was performed using in-built Mann-Whitney U test.

13. Digital Light Processing 3D Printing and Peptide Crosslinking

A new set of peptides (DGREFEFKa and KFKFKa), where Ka stands for allyloxycarbonyl (alloc) sidechain to crosslink with methacrylated gelatin (GelMA) using a 405 nm light source, were designed and synthesized as mentioned in Section 2-*Peptide synthesis*. Cantilever substrates were fabricated using a BIONOVAX digital light processing (DLP) printer (CELLINK). Designs were created in Fusion 360 and converted into PNG image slices using the slicer software integrated within the BIONOVAX system. The construct featured a central cantilever structure enclosed within a 7 × 7 × 1 mm (length × width × height) well. Each cantilever measured 5.7 × 5.7 × 0.15 mm and incorporated a microgroove surface pattern composed of trapezoidal ridges with a 100 µm bottom base width, 20 µm top base width, and 100 µm height (Figure S24). Constructs were printed using a 10% (w/v) methacrylated gelatin (GelMA; Advanced Biomatrix catalog # 5272) solution containing 0.5% (w/v) lithium phenyl-2,4,6-trimethylbenzoylphosphinate (LAP; Advanced Biomatrix catalog # 5272), both dissolved in 1X PBS. Printing was performed under 405 nm light. Following fabrication, constructs were washed with 1X PBS at 37 °C to wash away residual GelMA. KFKFKa-PDI and DGREFEFKa-4T were dissolved in UltraPure distilled water with a concentration of 1 mM for KFKFKa-PDI and 2 mM for DGREFEFKa-4T. The constructs were crosslinked for 5 min using 405 nm light after 20 µL of KFKFKa-PDI were dropcasted to cover the constructs. After 5 min, the constructs were washed once with 1X PBS. Then, 20 µL of DGREFEFKa-4T were dropcasted on top of the constructs and crosslinked for 5 min using the same light source. The constructs were then washed once with 1X PBS. Then, the constructs were incubated in the filtered Penicillin G potassium salt (Sigma Aldrich, catalog # P7794-100MU) dissolved in sterile, UltraPure distilled water (10,000 MU/mL) for 30 min, followed by 3 times washing with 1X sterile PBS. Lastly, the constructs were coated with fibronectin at 50 µg/mL overnight. Before seeding NRVMs, fibronectin coating was removed from the constructs and washed 3 times with 1X sterile PBS. NRVMs were seeded on the constructs at a seeding density of 1.5 × 10^6 cells/cm^2. NRVMs were maintained

using M199 medium supplemented with glucose, L-glutamine, vitamin B_{12}, HEPES, MEM-α, and 30% FBS on day 1, and the same media supplemented with 10% FBS on day 2. On day 4, the constructs with NRVMs were checked under the microscope to confirm the health and adhesion of the cells to the constructs to control the quality of the samples. The constructs that met the selection criteria were chosen to be further stimulated using the same light stimulation set up described in "Device fabrication and photocurrent measurement" 415-nm fiber-coupled LED light source. The frequency was introduced using a programmable pulse generator (Prizmatix Pulser). During the measurements, the samples were incubated in the 3 mL of warm, sterile, Tyrode's solution (119 mM NaCl, 5 mM KCl, 25 mM HEPES pH 7.4, 2 mM $CaCl_2$, 2 mM $MgCl_2$, 6 g/L glucose in 500 mL MilliQ water) and the temperature was maintained using automatic temperature controller (Warner Instruments; Catalog # TC-324C) and a stage adapter (Warner Instruments; Catalog # SA-20MW-AL-N). The videos were recorded using an Olympus SZ61 stereomicroscope attached with FL-W Color Correction filter (52 mm, Hoya Filter) and EPView software. The data was processed using Fiji software.

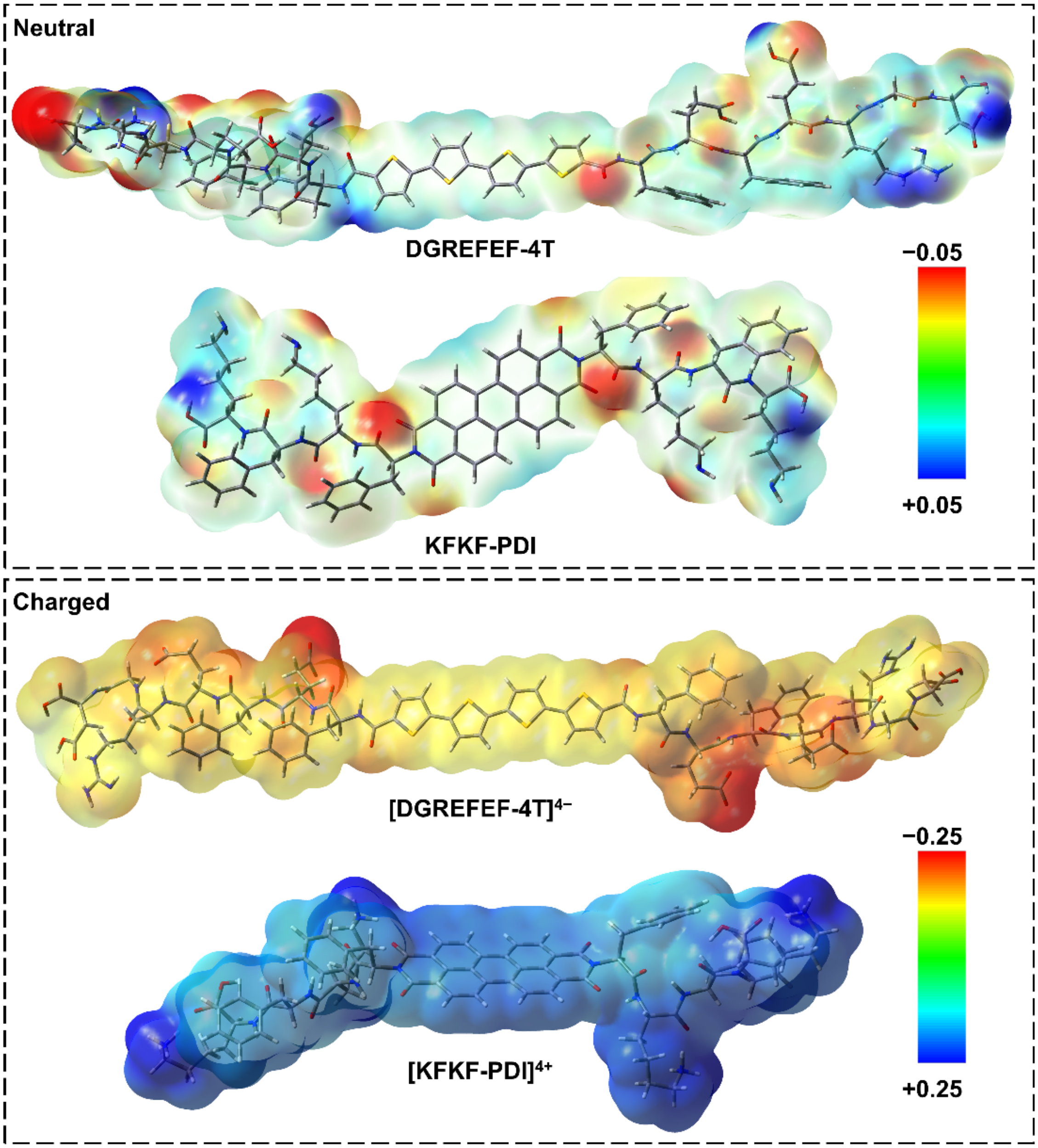

Figure S1. Electrostatic potential (ESP) maps of designed peptides showing the electrostatic interactions of the two main components of the coassembly system studied here.

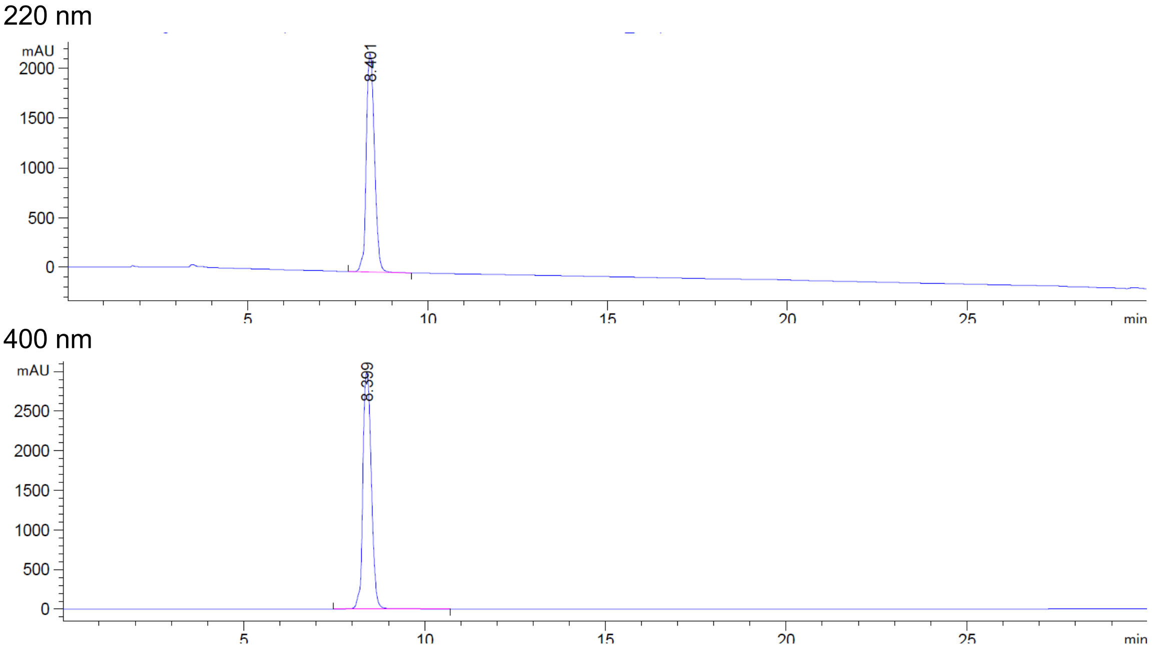

Figure S2. Analytical HPLC profile of DGREFEF-4T monitored at 220 and 400 nm.

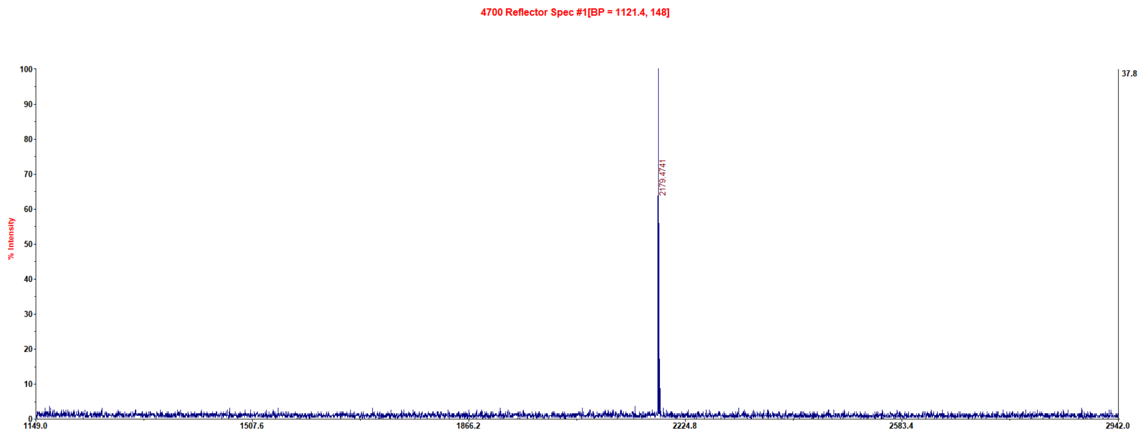

Figure S3. MALDI profile of DGREFEF-4T, m/z found: 2179.4741, calculated: 2179.6926.

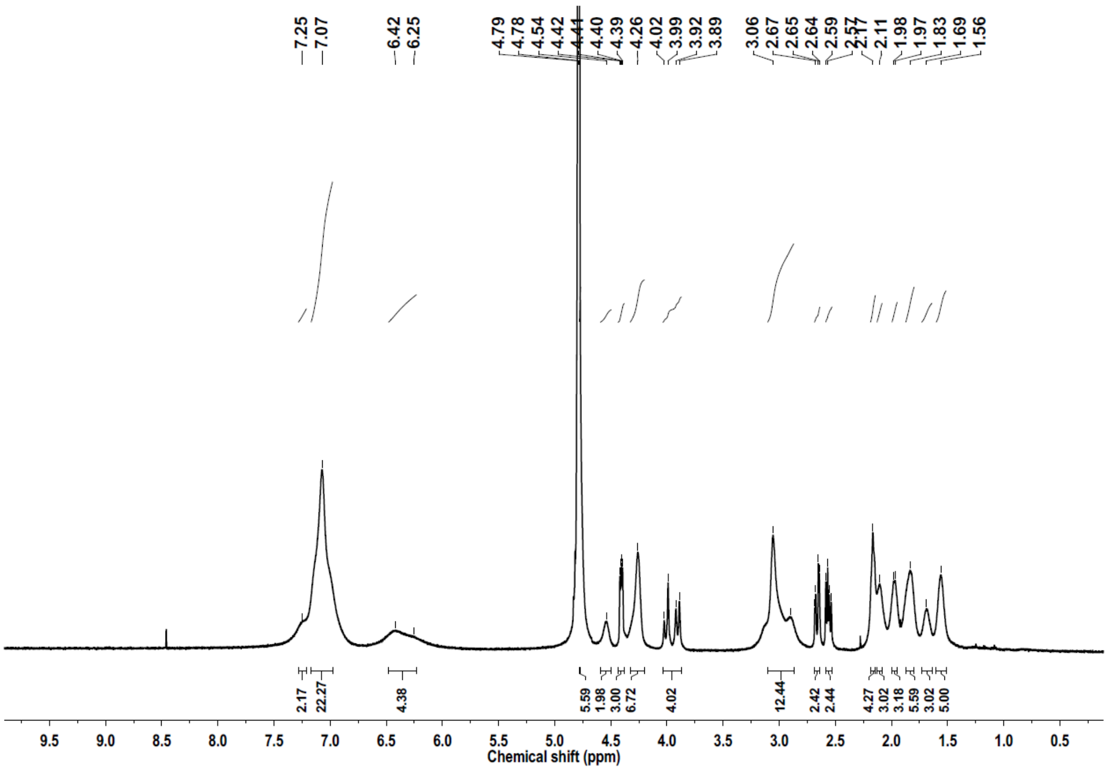

Figure S4. ^{1}H-NMR spectra (D_2O) of DGREFEF-4T. Peak assignments are detailed in Section 2 of this document, page 3.

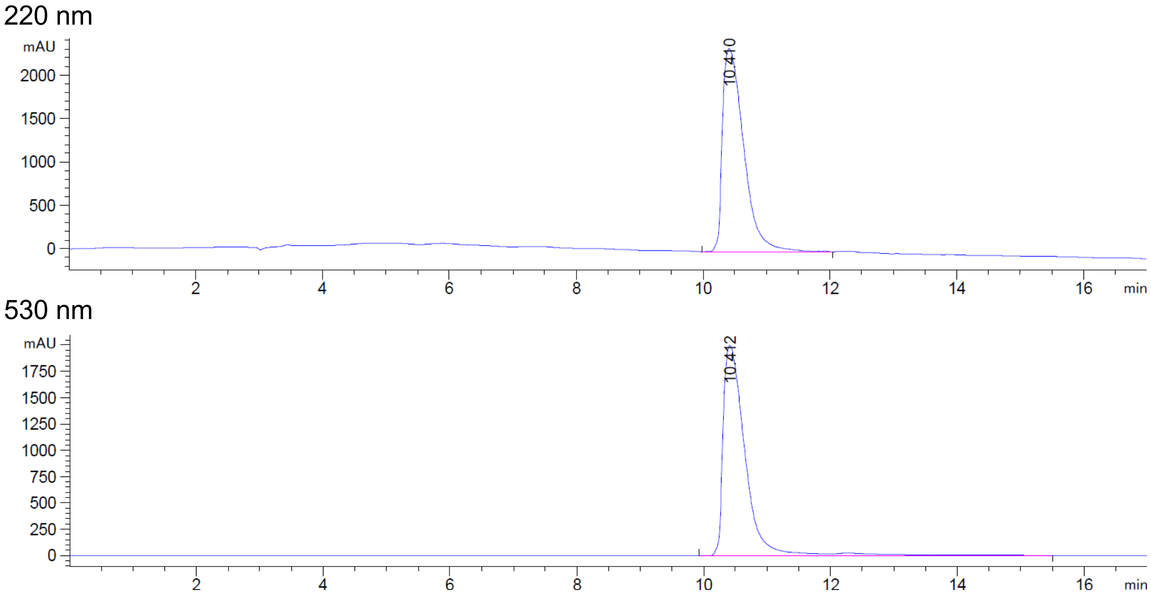

Figure S5. Analytical HPLC profile of KFKF-PDI monitored at 220 and 530 nm.

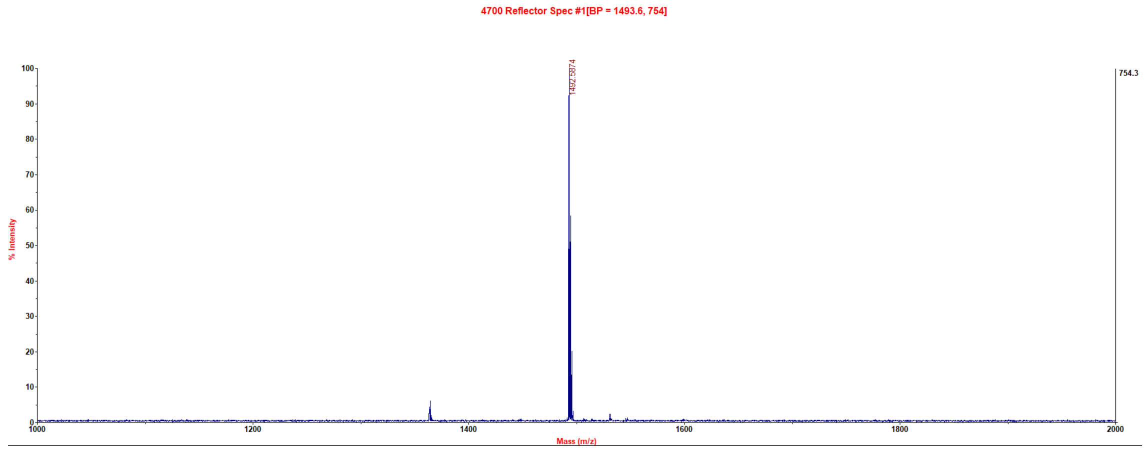

Figure S6. MALDI profile of KFKF-PDI, m/z found: 1492.5874, calculated: 1492.6856.

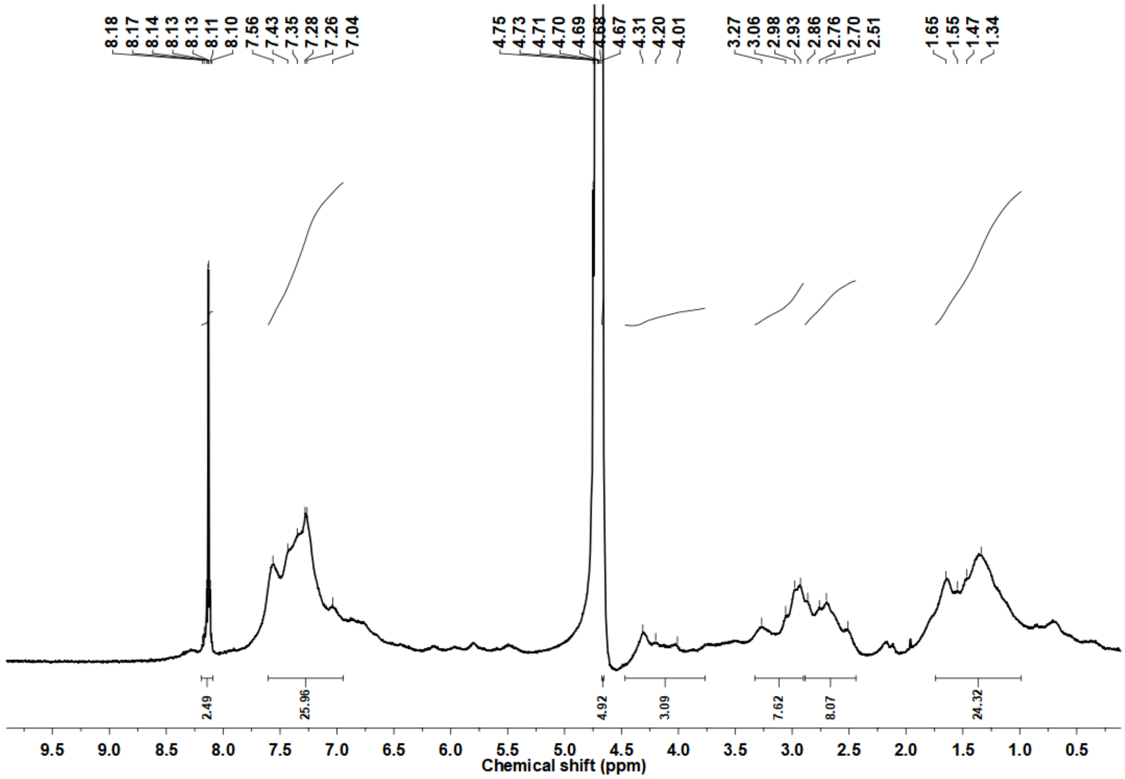

Figure S7. ^{1}H-NMR spectra (D_2O) of KFKF-PDI. Peak assignments are detailed in Section 2 of this document, page 3.

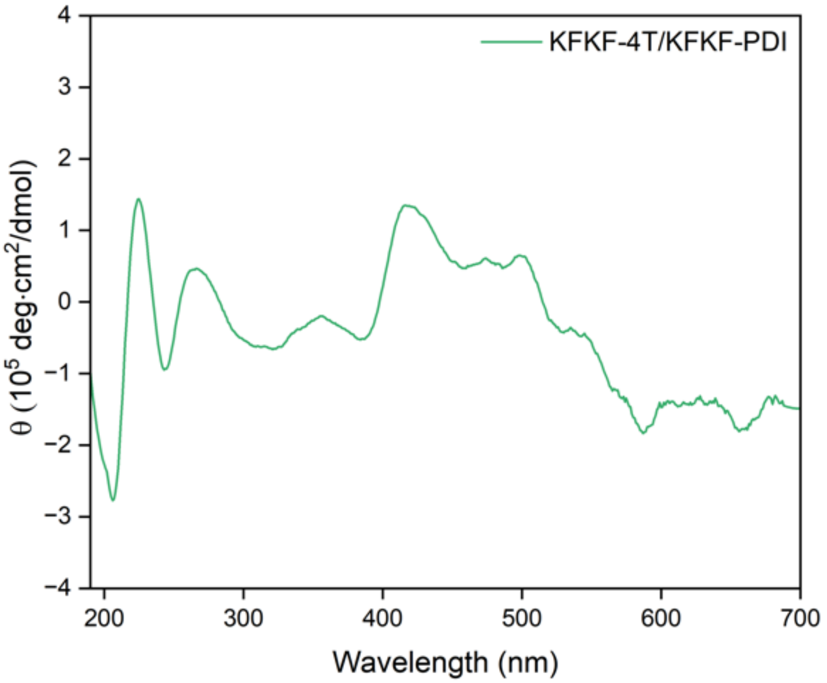

Figure S8. Circular dichroism spectrum of KFKF-4T/KFKF-PDI coassembly solution.

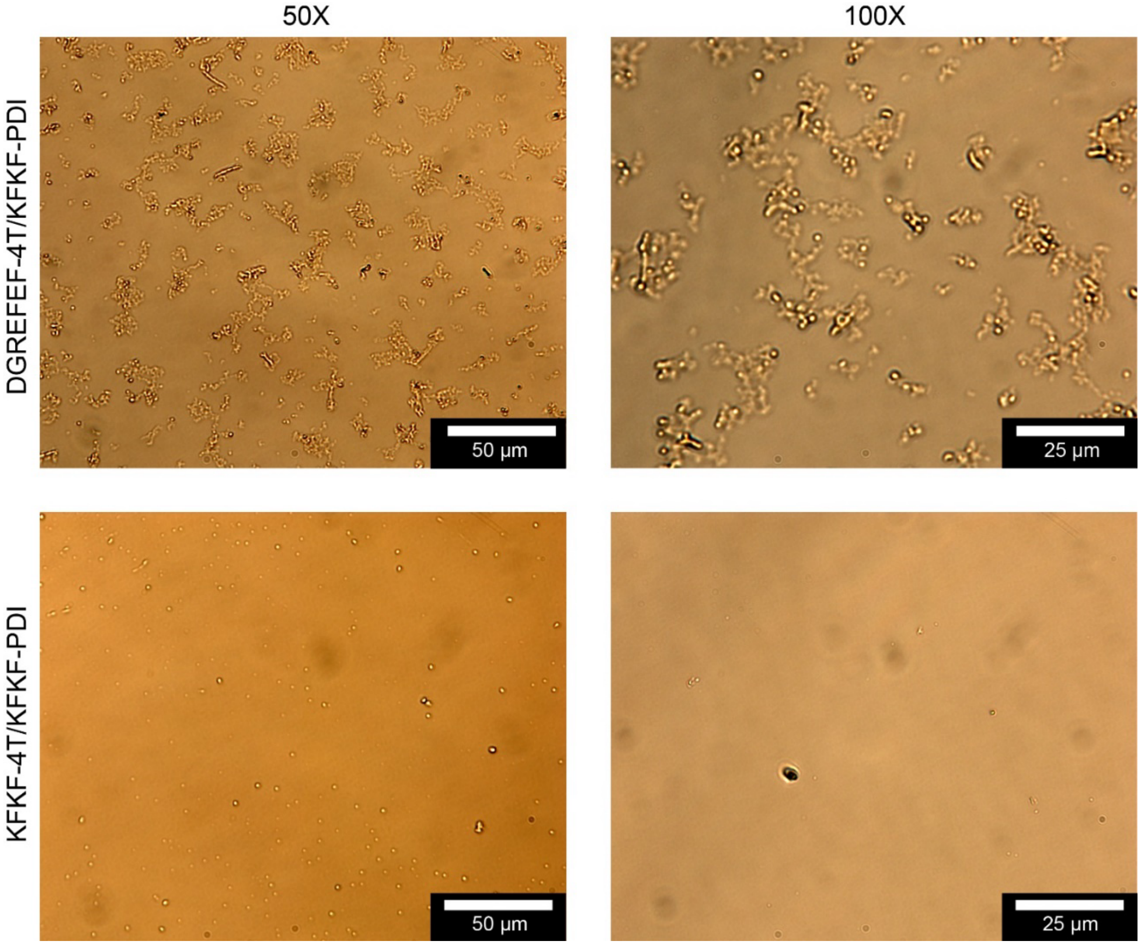

Figure S9. Representative images of charge complementary peptide-donor/acceptor coassembly (DGREFEF-4T/KFKF-PDI) and sequence-matched peptide-donor/acceptor coassembly (KFKF-4T/KFKF-PDI) at 50× and 100× magnifications; taken via optical microscopy in transmission mode.

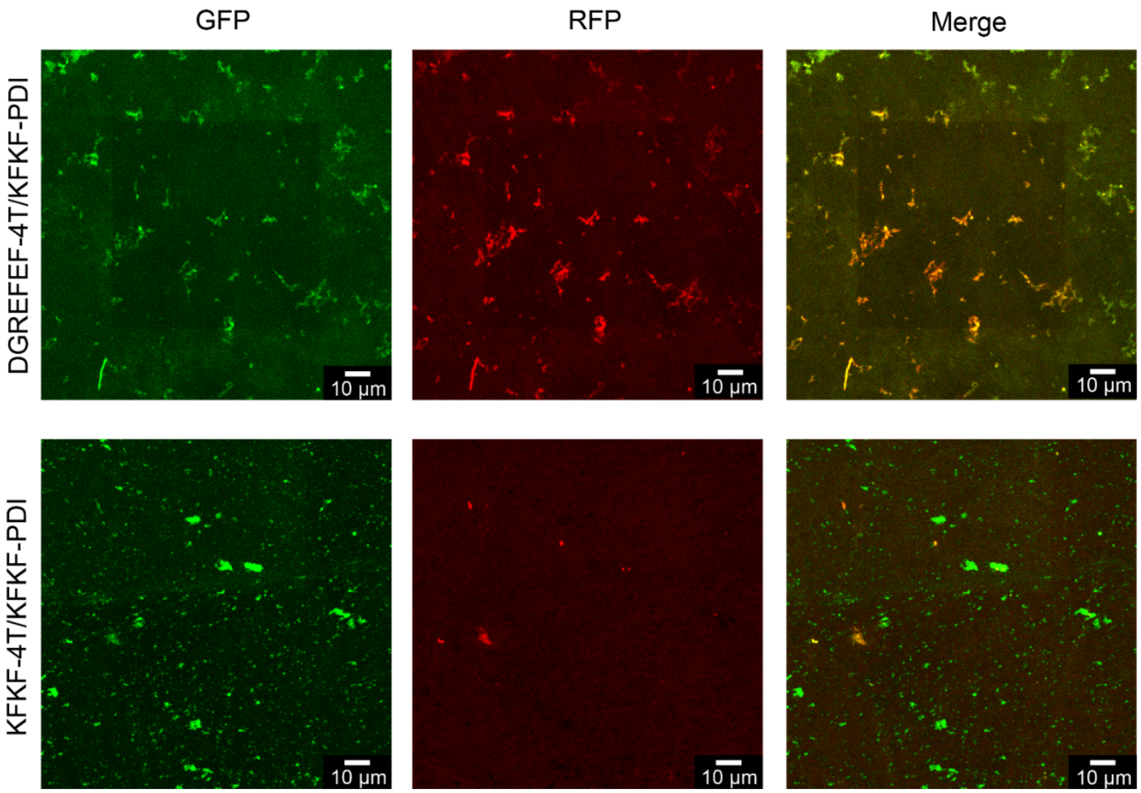

Figure S10. Fluorescence images of a charge complementary peptide-donor/acceptor coassembly (DGREFEF-4T/KFKF-PDI) and a sequence-matched peptide-donor/acceptor coassembly (KFKF-4T/KFKF-PDI) were visualized under GFP and RFP channels. GFP was used to monitor 4T signals and RFP was used to monitor PDI signals.

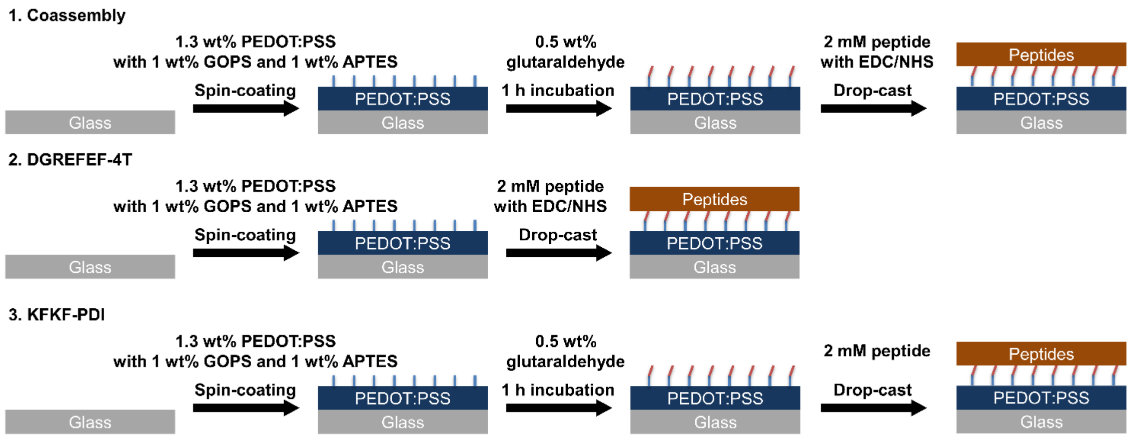

Figure S11. Fabrication process of peptide assembled films.

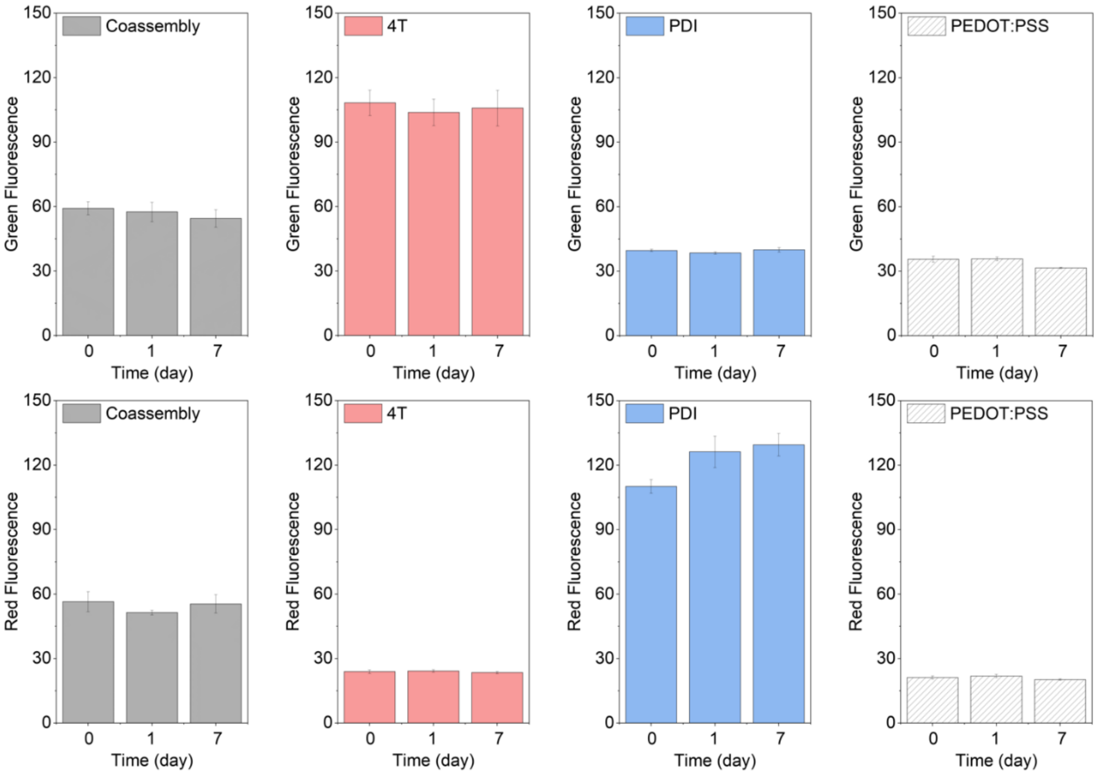

Figure S12. Stability of assembled peptide films plotted by the fluorescence immersed in PBS (1X) solution. The prepared samples were immersed into PBS (1X) solution and naturally dried before absorption measurement using a fluorescence microscope.

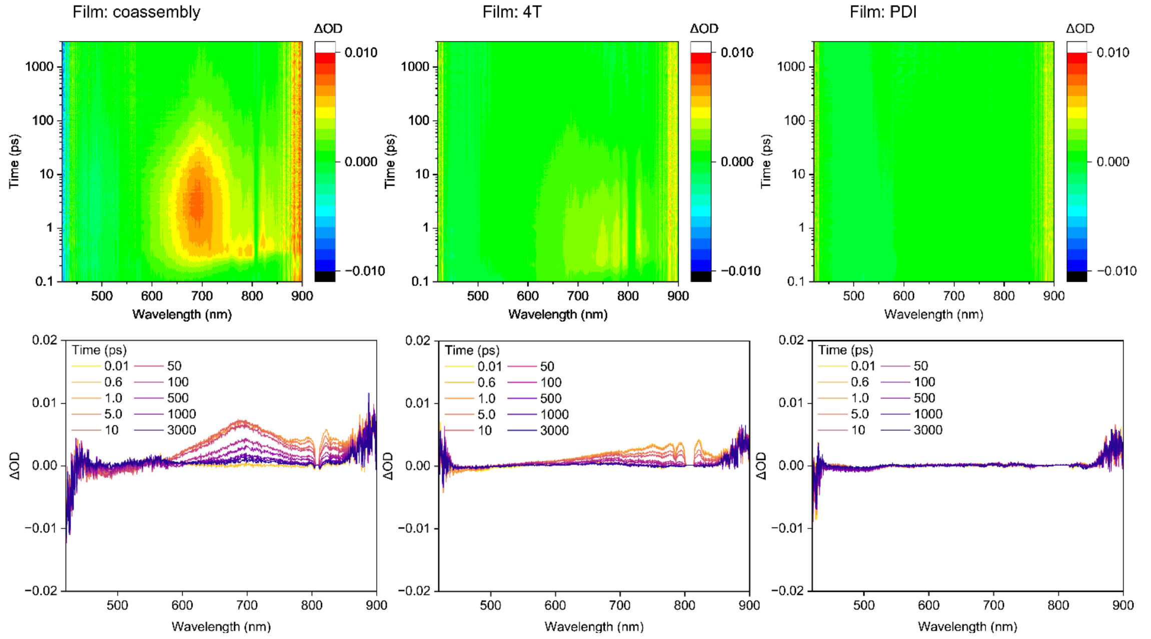

Figure S13. Transient absorption (TA) spectra patterns and profiles of peptide assemblies in films of coassembly, DGREFEF-4T, and KFKF-PDI. Excitation wavelength: 400 nm.

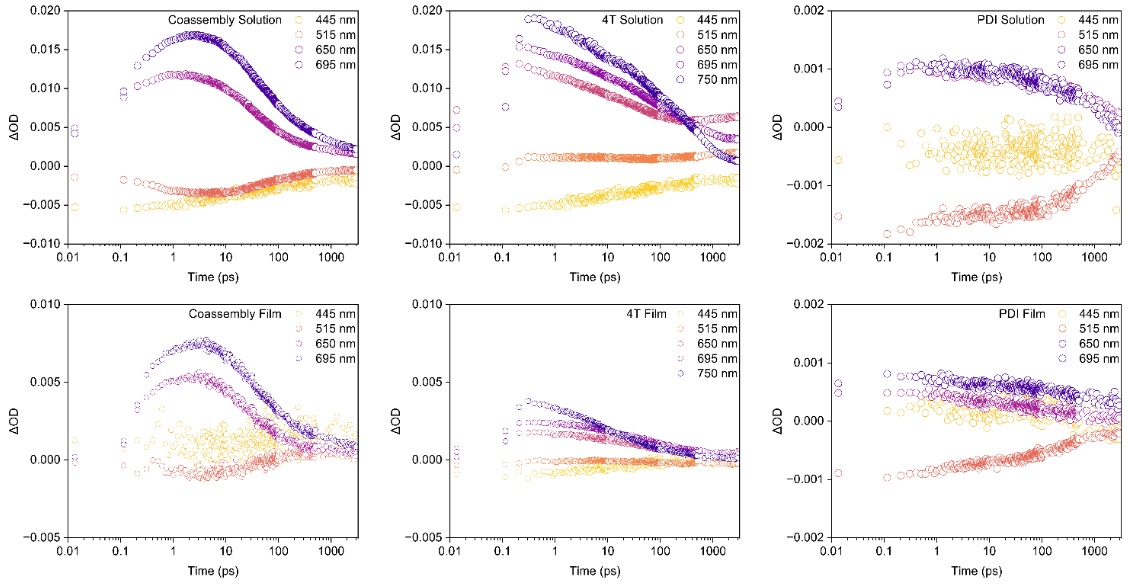

Figure S14. TA intensity decay of peptide assemblies at difference wavelength in solution or film state with Tyrode's solution.

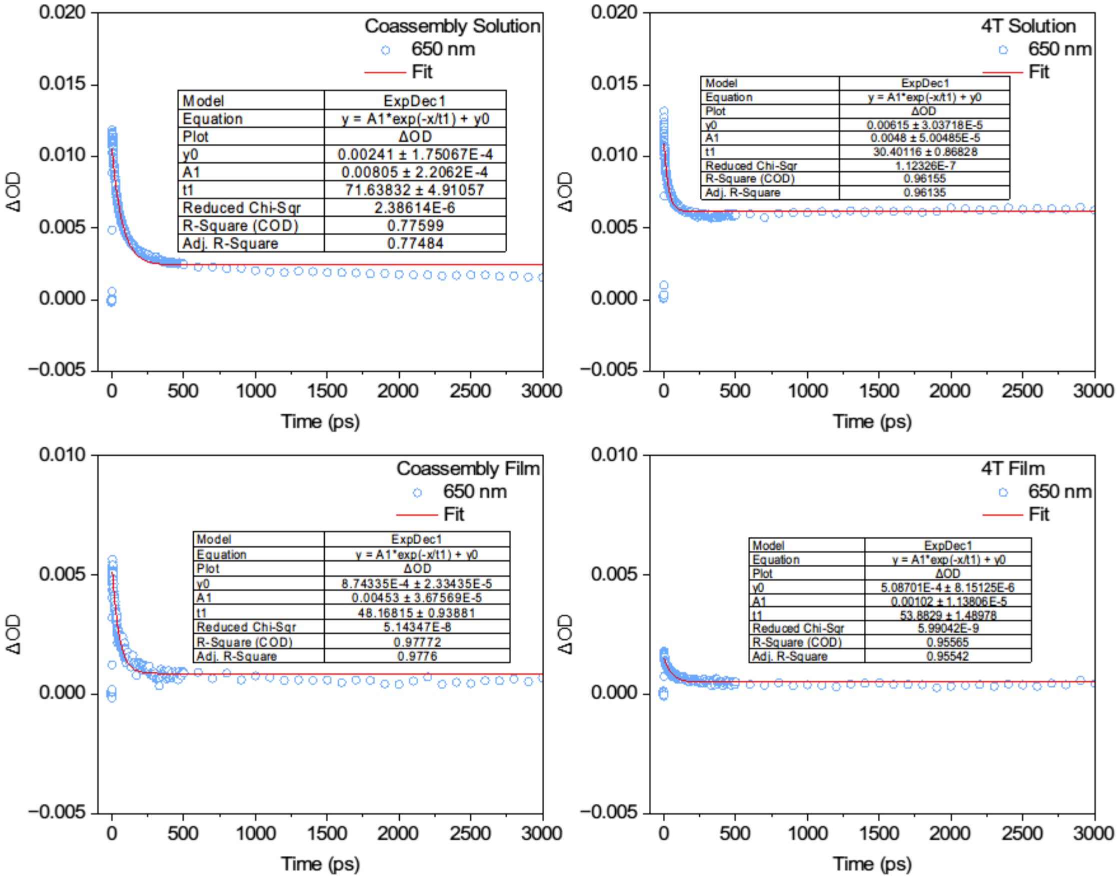

Figure S15. TA intensity decay and exponential decay fitting of peptide assemblies at 650 nm of peptide assemblies in solution or film state with Tyrode's solution.

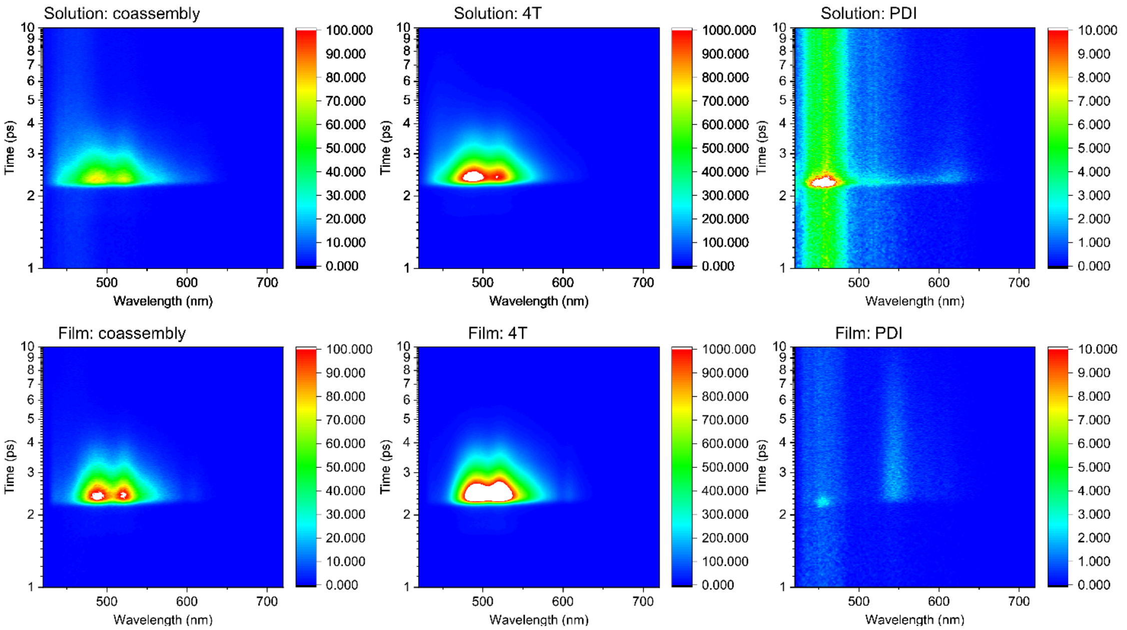

Figure S16. Time-resolved photoluminescence (TRPL) patterns of peptide assemblies in solution or film state with Tyrode’s solution.

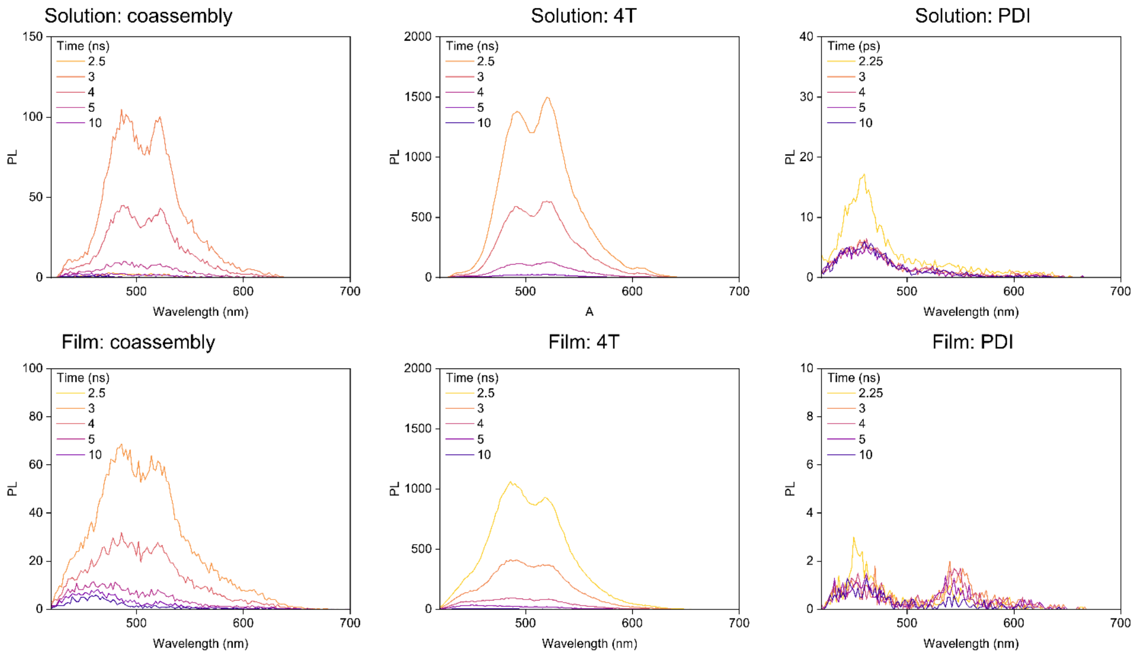

Figure S17. Time-resolved photoluminescence (TRPL) profiles of peptide assemblies in solution or film state with Tyrode's solution.

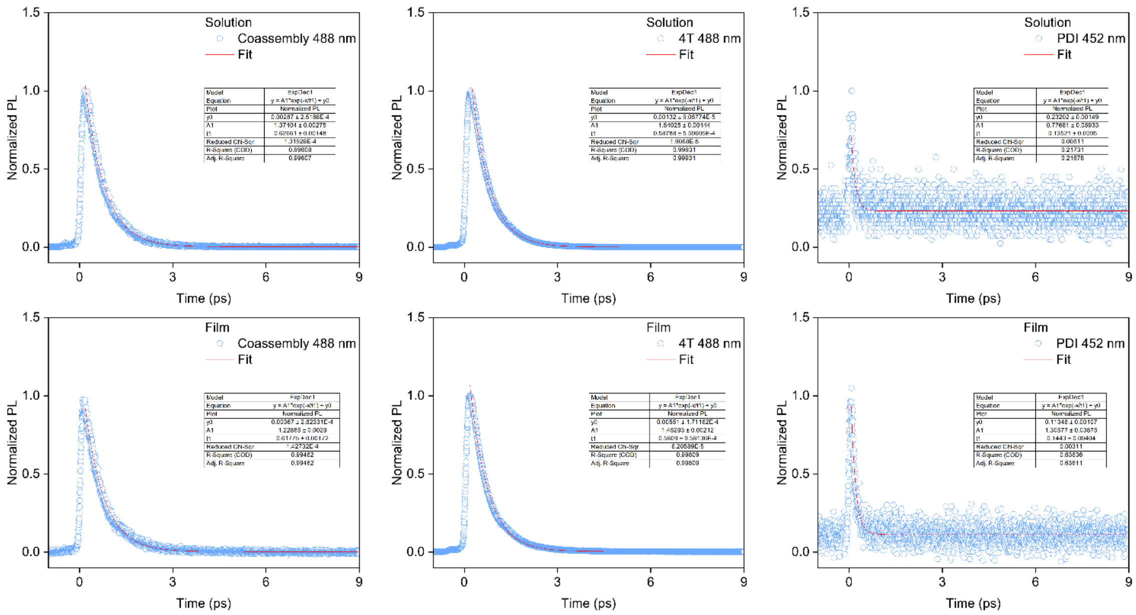

Figure S18. Time-resolved photoluminescence (TRPL) intensity decay at the maximum intensity and exponential decay fitting of peptide assemblies in solution or film state with Tyrode's solution.

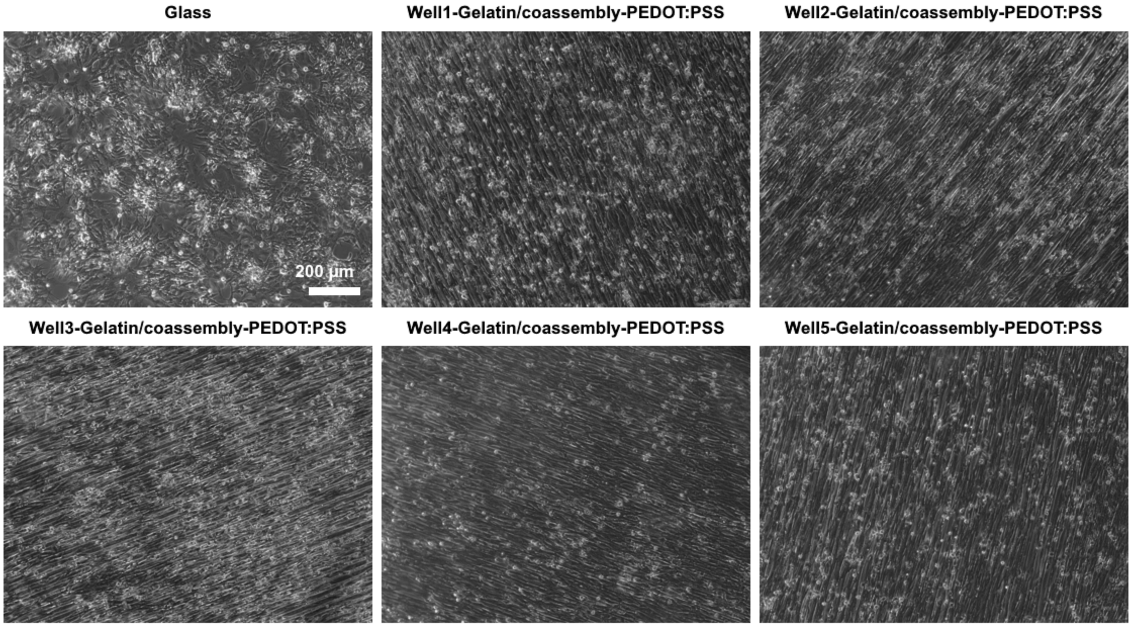

Figure S19. Supplementary brightfield microscope images of NRVMs on glass as control and aligned NRVMs on patterned gelatin mixed coassembly.

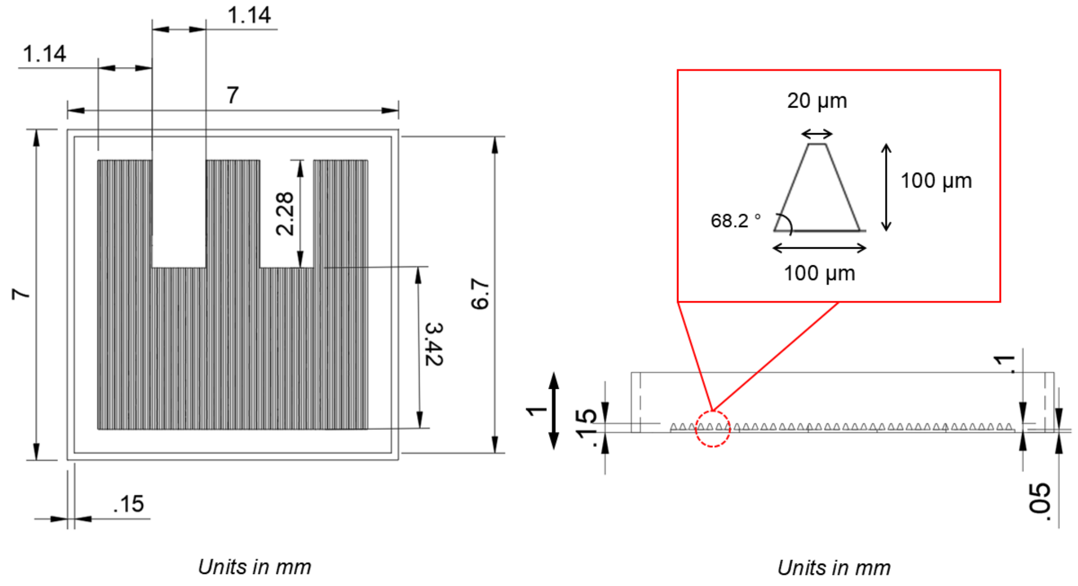

Figure S20. Schematic illustration of a multi-cantilever design for fabricating cardiac muscular actuators in vitro.

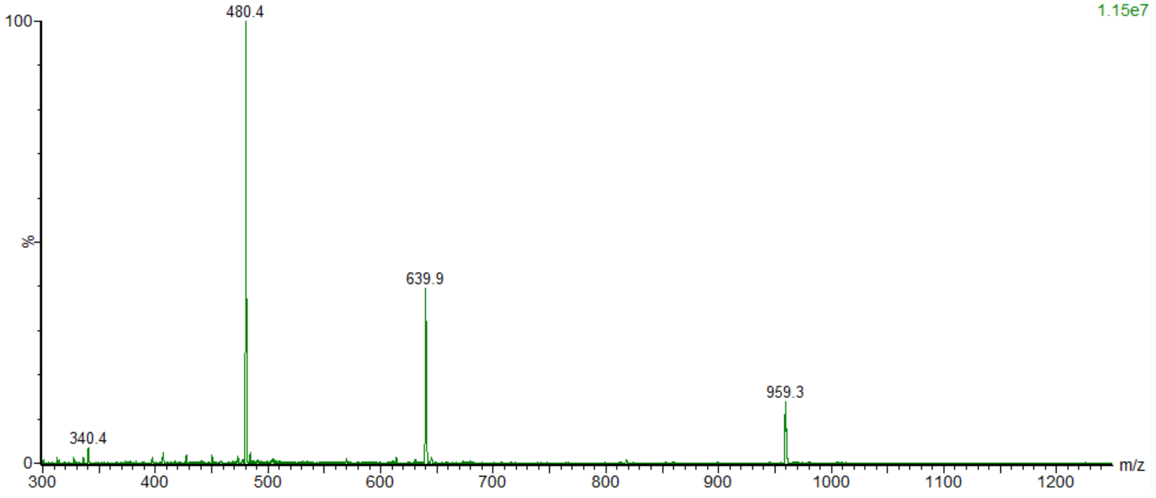

Figure S21. ESI-MS spectrum of KFKFKa-PDI. m/z found $[M+2H]^{2+}/2$: 959.3, $[M+3H]^{3+}/3$: 639.9, $[M+4H]^{4+}/4$: 480.4, calculated $[M+2H]^{2+}/2$: 959.5, $[M+3H]^{3+}/3$: 640.3, $[M+4H]^{4+}/4$: 480.5.

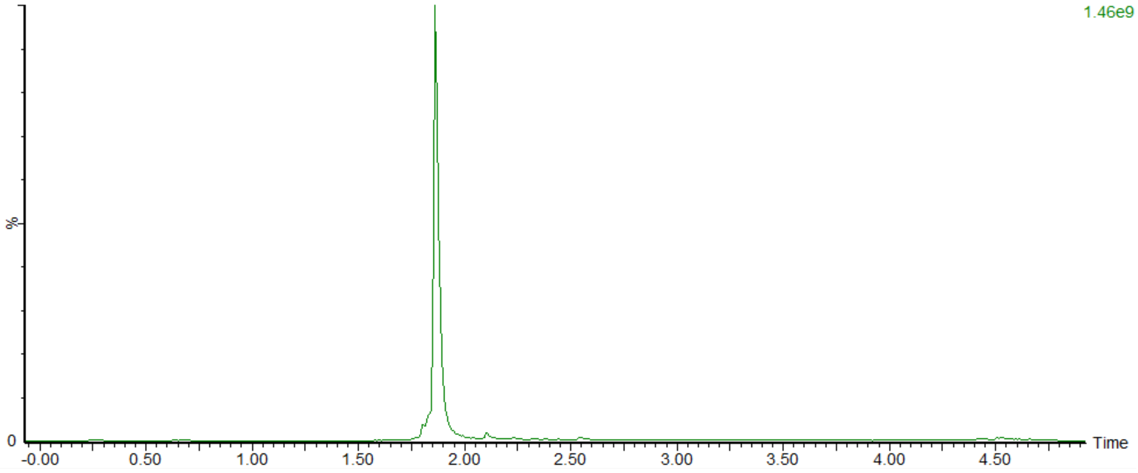

Figure S22. UPLC trace of KFKFKa-PDI monitored for the purity at 200-500 nm.

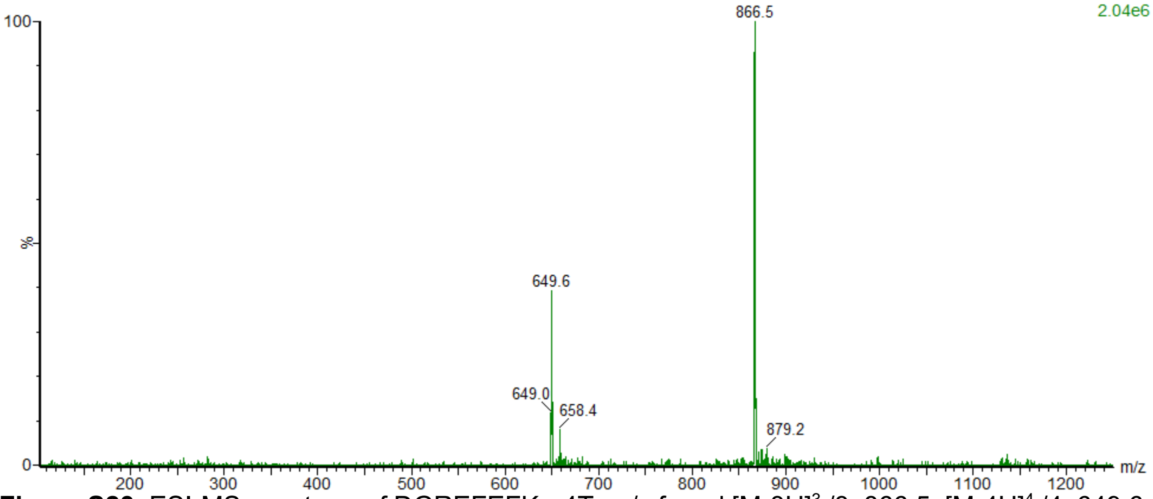

Figure S23. ESI-MS spectrum of DGREFEFKa-4T, m/z found $[M-3H]^{3-}/3$: 866.5, $[M-4H]^{4-}/4$: 649.6, calculated $[M-3H]^{3-}/3$: 866.9, $[M-4H]^{4-}/4$: 649.9.

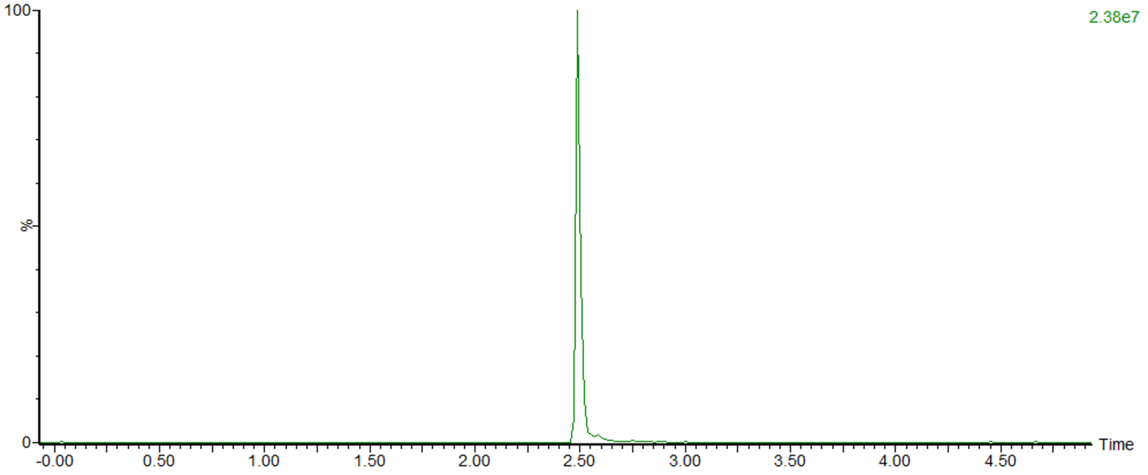

Figure S24. UPLC trace of DGREFEFKa-4T monitored for the purity at 200-500 nm.

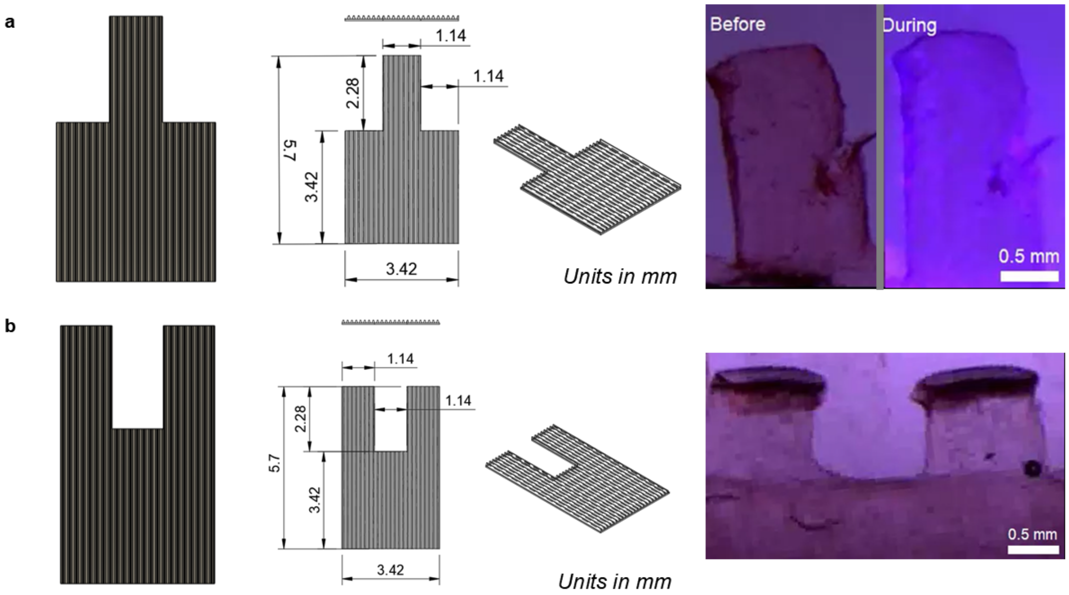

Figure S25. A schematic of modified cantilever designs and DLP-printed constructs with NRVMs. (a) NRVMS on DLP-printed constructs before and during stimulation and its corresponding schematic diagram. (b) Schematic diagram of an NRVM-seeded DLP-printed construct demonstrating actuation behavior. Representative still images from Supplementary Videos 7 and 8 are shown. Scale bar= 0.5 mm.

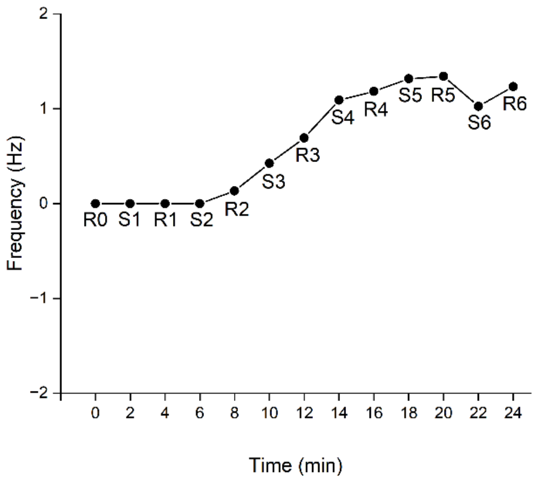

Figure S26. Representative contraction frequency (beat rate) response for a muscular thin film actuator functionalized with optoelectronic peptide layers. The “on” (S; stimulation period) and “off” (R; resting period) cycles of light stimulation of NRVMs on DLP printed cantilevers at different time points.

Movie S1. Video of NRVMs interfaced with coassembled KFKF-PDI/DGREFEF-4T on PEDOT:PSS film before light stimulation.

Movie S2. Video of NRVMs interfaced with coassembled KFKF-PDI/DGREFEF-4T on PEDOT:PSS film during light stimulation of 415 nm with 2 Hz pulsing frequency.

Movie S3. Video of NRVMs interfaced with coassembled KFKF-PDI/DGREFEF-4T on PEDOT:PSS film before light stimulation.

Movie S4. Video of NRVMs interfaced with coassembled KFKF-PDI/DGREFEF-4T on PEDOT:PSS film during light stimulation of 415 nm with 2 Hz pulsing frequency.

Movie S5. Video of NRVMs on glass control substrate before light stimulation.

Movie S6. Video of NRVMs on glass control substrate during light stimulation of 415 nm with 2 Hz pulsing frequency.

Movie S7. Video of NRVMs on a DLP-printed cantilever before and during (t=17 min) light stimulation of 415 nm with 2 Hz pulsing frequency.

Movie S8. Video of NRVMs on a DLP-printed cantilever after 2 min of light stimulation of 415 nm with 2 Hz pulsing frequency, showing an actuating cardiac biohybrid construct that bears an interfacial layer of photocurrent-generating peptides.

SI References

1. A. Demessence, *et al.*, Synthesis, optical and magnetic properties of hybrid α,α′-oligothiophenecarboxylates/transition metal hydroxide multilayered compounds. *J. Mater. Chem.* **20**, 9401 (2010).
2. Z.-F. Yao, Y. Kuang, P. Kohl, Y. Li, H. A. M. Ardoña, Carbodiimide-fueled assembly of π-conjugated peptides regulated by electrostatic interactions. *ChemSystemsChem* **5**, e202300003 (2023).
3. H. A. M. Ardoña, K. Besar, M. Togninalli, H. E. Katz, J. D. Tovar, Sequence-dependent mechanical, photophysical and electrical properties of pi-conjugated peptide hydrogelators. *J. Mater. Chem. C* **3**, 6505–6514 (2015).
4. E. M. Lundqvist, *et al.*, Micropatterning photoconductive peptide assemblies on stiff and soft biomaterial substrates. *ACS Appl. Mater. Interfaces* **17**, 31982–31992 (2025).
5. S. Grimme, C. Bannwarth, P. Shushkov, A robust and accurate tight-binding quantum chemical method for structures, vibrational frequencies, and noncovalent interactions of large molecular systems parametrized for all spd-block elements (Z = 1–86). *J. Chem. Theory Comput.* **13**, 1989–2009 (2017).
6. C. F. MacRae, *et al.*, Mercury 4.0 : from visualization to analysis, design and prediction. *J. Appl. Crystallogr.* **53**, 226–235 (2020).
7. T. Lu, F. Chen, Multiwfn: A multifunctional wavefunction analyzer. *J. Comput. Chem.* **33**, 580–592 (2012).
8. C. R. Morcombe, K. W. Zilm, Chemical shift referencing in MAS solid state NMR. *J. Magn. Reson.* **162**, 479–486 (2003).
9. A. Pines, M. G. Gibby, J. S. Waugh, Proton-enhanced NMR of dilute spins in solids. *J. Chem. Phys.* **59**, 569–590 (1973).
10. E. . Stejskal, J. Schaefer, J. . Waugh, Magic-angle spinning and polarization transfer in proton-enhanced NMR. *J. Magn. Reson.* **28**, 105–112 (1977).
11. J. Ilavsky, P. R. Jemian, Irena : tool suite for modeling and analysis of small-angle scattering. *J. Appl. Crystallogr.* **42**, 347–353 (2009).
12. J. L. P. Morival, H. P. Widyastuti, C. H. H. Nguyen, M. V. Zaragoza, T. L. Downing, DNA methylation analysis reveals epimutation hotspots in patients with dilated cardiomyopathy-associated laminopathies. *Clin. Epigenetics* **13**, 139 (2021).
13. X. Lian, *et al.*, Directed cardiomyocyte differentiation from human pluripotent stem cells by modulating Wnt/β-catenin signaling under fully defined conditions. *Nat. Protoc.* **8**, 162–175 (2013).
14. M. V. Zaragoza, *et al.*, LMNA-related dilated cardiomyopathy: single-cell transcriptomics during patient-derived iPSC differentiation support cell type and lineage-specific dysregulation of gene expression and development for cardiomyocytes and epicardium-derived cells with lamin. *Cells* **13**, 1479 (2024).
